# Ablation of cardiomyocyte-derived BDNF during development causes myocardial degeneration and heart failure in the adult mouse heart

**DOI:** 10.3389/fcvm.2022.967463

**Published:** 2022-08-18

**Authors:** Lilin Li, Hongyan Guo, Binglin Lai, Chunbao Liang, Hongyi Chen, Yilin Chen, Weimin Guo, Ziqiang Yuan, Ruijin Huang, Zhaohua Zeng, Liying Liang, Hui Zhao, Xin Zheng, Yanmei Li, Qin Pu, Xufeng Qi, Dongqing Cai

**Affiliations:** ^1^Key Laboratory of Regenerative Medicine, Ministry of Education, Jinan University, Guangzhou, China; ^2^Joint Laboratory for Regenerative Medicine, Chinese University of Hong Kong-Jinan University, Guangzhou, China; ^3^International Base of Collaboration for Science and Technology (JNU), The Ministry of Science and Technology and Guangdong Province, Guangzhou, China; ^4^Department of Developmental and Regenerative Biology, Jinan University, Guangzhou, China; ^5^Jiangxi Provincial Key Laboratory of Medical Immunology and Immunotherapy, Jiangxi Academy of Medical Sciences, Nanchang, China; ^6^Department of Medical Oncology, Robert Wood Johnson of Medical School, Cancer Institute of New Jersey, New Brunswick, NJ, United States; ^7^Department of Neuroanatomy, Institute of Anatomy, University of Bonn, Bonn, Germany; ^8^Department of Anatomy and Molecular Embryology, Institute of Anatomy and Cell Biology, University of Freiburg, Freiburg, Germany; ^9^Division of Cardiology, Department of Internal Medicine, The First Affiliated Hospital of Guangzhou Medical University, Guangzhou, China; ^10^Stem Cell and Regeneration TRP, School of Biomedical Sciences, Chinese University of Hong Kong, Shatin, Hong Kong SAR, China

**Keywords:** cardiomyocyte, cardiomyocyte-derived BDNF conditional knockout, heart failure, degeneration of the myocardium, myocardial infarction

## Abstract

**Objective:**

Brain-derived neurotrophic factor (BDNF) and its receptor TrkB-T1 were recently found to be expressed in cardiomyocytes. However, the functional role of cardiomyocyte-derived BDNF in heart pathophysiology is not yet fully known. Recent studies revealed that BDNF-TrkB pathway plays a critical role to maintain integrity of cardiac structure and function, cardiac pathology and regeneration of myocardial infarction (MI). Therefore, the BDNF-TrkB pathway may be a novel target for myocardial pathophysiology in the adult heart.

**Approach and results:**

In the present study, we established a cardiomyocyte-derived BDNF conditional knockout mouse in which BDNF expression in developing cardiomyocytes is ablated under the control of the Myosin heavy chain 6 (MYH6) promoter. The results of the present study show that ablation of cardiomyocyte-derived BDNF during development does not impair survival, growth or reproduction; however, in the young adult heart, it causes cardiomyocyte death, degeneration of the myocardium, cardiomyocyte hypertrophy, left atrial appendage thrombosis, decreased cardiac function, increased cardiac inflammation and ROS activity, and metabolic disorders, leading to heart failure (HF) in the adult heart and eventually resulting in a decrease in the one-year survival rate. In addition, ablation of cardiomyocyte-derived BDNF during the developmental stage leads to exacerbation of cardiac dysfunction and poor regeneration after MI in adult hearts.

**Conclusion:**

Cardiomyocyte-derived BDNF is irreplaceable for maintaining the integrity of cardiac structure and function in the adult heart and regeneration after MI. Therefore, the BDNF-TrkB pathway will be a novel target for myocardial pathophysiology in the adult heart.

## Introduction

The brain-derived neurotrophic factor (BDNF) pathway is highly active in the nervous system and plays critical roles in promoting growth, survival, synaptic connection, neuronal repair and regeneration of the nervous system as well as the treatment of neurological diseases (1–3). In addition, BDNF also plays important roles in non-neuronal tissues, including vasculature (4), platelets (5), vascular smooth muscle cells and macrophages (6, 7), early hematopoietic cells (8, 9), activated lymphocytes (10) monocytes (11), and skeletal muscle (12).

Among the notable progresses related to BDNF-TrkB signaling, the BDNF-TrkB pathway plays a key role in the pathophysiology and regeneration of the cardiovascular system. BDNF and its receptor TrkB are expressed in the endothelial cells of the coronary arteries (13) and are known to be associated with capillary development and cardiac endothelium formation (14, 15) in heart tissue during the late gestation period (7). BDNF has been reported to play an important role in diminished endothelial cell-cell contact and endothelial cell apoptosis in mouse embryonic intramyocardial arteries, capillaries, and the heart, while BDNF knockout leads to intraventricular wall hemorrhage and perinatal lethality (13, 16–18). Overexpression of BDNF in the midgestational mouse heart results in an increase in capillary density (18). Separately, TrkB^–/–^ mice showed a marked reduction in blood vessel density and an increased number of apoptotic endothelial cells, predominantly in the subepicardial region of the developing heart (17). Recently, we demonstrated that BDNF promoted the migration of young cardiac microvascular endothelial cells (CMECs) *via* activation of the BDNF-TrkB-FL-PI3K/Akt pathway, which may benefit angiogenesis after MI. However, the aging of CMECs led to changes in the expression of isoforms of the receptor TrkB: among the three isoforms of TrkB (TrkB-FL, TrkB-T1, and TrkB-T2), only the truncated TrkB-T1 isoform continued to be expressed, which led to dysfunction of its ligand, decreased CMEC migration, and increased injury in aging hearts. Although the efficacy of promoting migration via the BDNF-TrkB-T1 pathway in aged CMECs was significantly decreased compared with that in young CMECs via the BDNF-TrkB-FL pathway, the BDNF-TrkB-T1 pathway in aged CMECs was still able to promote the migration of aged CMECs (19). More recently, we further demonstrated that aged CMECs utilized BDNF-TrkB-T1 signaling to recruit Willin as a downstream effector to activate the Hippo pathway and then promote migration. This finding suggests that the aging process shifts aged CMECs that express TrkB-T1 receptors by transducing BDNF signals via the BDNF-TrkB-T1-Willin-Hippo pathway and that this change might be an important mechanism and therapeutic target behind the dysfunction of cardiac angiogenesis observed in aged hearts (20). All these findings clearly reveal that the BDNF-TrkB pathway plays an essential role in the development and growth of the vasculature as well as angiogenesis by maintaining the integrity and regenerative capacity of the cardiovascular system.

In addition to endothelial cells, recent progress has demonstrated that cardiomyocytes also express BDNF and its receptor TrkB-T1 (14, 21–23). BDNF-TrkB signaling is required for the heart to fully contract and relax. These actions occur independently from and in addition to β-adrenergic influence. BDNF-induced enhancement of myocardial performance occurs via direct modulation of Ca^2+^ cycling in a calmodulin-dependent protein kinase II-dependent manner (23). It was reported that BDNF regulates the cardiac contraction force independent of nervous system innervation. This function is mediated by the truncated TrkB-T1 receptor expressed in cardiomyocytes. Loss of TrkB-T1 in these cells impairs calcium signaling and causes cardiomyopathy (14). These findings suggest that cardiomyocyte-derived BDNF plays a critical role in cardiomyocyte contraction. In addition, it has been reported that homozygous systemic BDNF knockout mice die due to Heart failure (HF) in the fetal period (18). In hearts from an adult tamoxifen-induced systemic BDNF deletion myocardial infarction (MI) model, decreased cardiac function and myocardial angiogenesis in the infarct border zone, decreased expression of the prosurvival molecule Bcl-2 and increased infarct size, cardiomyocyte apoptosis and expression of the proapoptotic molecule Bax were found compared with those in wild-type MI hearts. These results suggested that systemic deletion of BDNF leads to exacerbation of cardiac dysfunction after MI. However, heart size and cardiac function were found at basal levels in tamoxifen-induced 2-month-old cardiomyocyte-specific BDNF conditional knockout mice. Ablation of BDNF in cardiomyocytes did not affect cardiac remodeling, cardiac function, myocardial angiogenesis or infarct size after MI. In contrast, in 2-month-old cardiomyocyte-specific inducible TrkB conditional knockout MI mouse model hearts, decreased cardiac function and myocardial angiogenesis in the infarct border zone and increased infarct size and cardiomyocyte apoptosis were found compared with those in wild-type MI hearts (22). The above findings combined with the inconsistent pathological outcomes observed in the adult inducible BDNF and TrkB conditional knockout models suggest that endogenous BDNF from cardiomyocytes plays an important role in regulating and maintaining cardiac pathophysiology. However, to date, whether cardiomyocyte-derived BDNF is irreplaceable for maintaining the integrity of cardiac structure and function in the heart and its exact functional role and molecular mechanism in cardiac pathophysiology are still unclear.

Owing to the limitation of the adult inducible cardiomyocyte-specific BDNF conditional knockout model, which only ablates BDNF from cardiomyocytes for a short amount of time in the adult heart, it is not an appropriate model for elucidating the importance of cardiomyocyte-derived BDNF in maintaining cardiac structure and function in dynamic scenarios from development to the adult stage. Therefore, the present study was designed to investigate the effects of ablating cardiomyocyte-derived BDNF during development on cardiac pathophysiology. Our results demonstrate that ablation of cardiomyocyte-derived BDNF during the development stage does not impair survival, growth or reproduction; however, in the young adult heart, it causes cardiomyocyte death, degeneration of the myocardium, cardiomyocyte hypertrophy, decreased cardiac function, increased cardiac inflammation and ROS activity, and metabolic disorders, leading to HF in the adult heart and eventually resulting in a decrease in the one-year survival rate. In addition, ablation of cardiomyocyte-derived BDNF during the developmental stage leads to exacerbation of cardiac dysfunction and poor regeneration after MI in adult hearts.

## Materials and methods

### Animals and generation of the cardiomyocyte-specific BDNF conditional knockout mouse

Stock BDNF (tm3Jae)/J mice (*BDNF^flox/flox^*; Stock #004339) and B6.FVB-Tg(Myh6-cre)2182Mds/J mice (Stock #011038) were obtained from the Jackson Laboratory. By expressing bacteriophage P1 Cre recombinase (Cre) in a tissue or cell-type specific manner, genes that are engineered with flanking loxP sites can be deleted or overexpressed in an analogous fashion. In the cardiac myocyte, this is most often achieved using Cre expression driven by the cardiac myocyte-specific alpha myosin heavy chain promoter (24, 25). Cre expression from this promoter has been shown to be both cardiac myocyte specific and to drive highly efficient recombination (24). In present study, in order to avoid the potential cardiotoxicity from Myh6-Cre, the mating strategy to generate Myh6-Cre-BDNF*^flox^* mice with mixed background was adopted (26, 27). The stock BDNF (tm3Jae)/J mice (*BDNF^flox/flox^*) were mated with the B6.FVB-Tg(Myh6-cre)2182Mds/J mice to obtain heterozygous cardiomyocyte-specific conditional BDNF knockout mice (Myh6-Cre^±^-BDNF*^flox/^*^Δ^) that were backcrossed to the *BDNF^flox/flox^* mice to obtain homozygous cardiomyocyte-specific conditional BDNF knockout mice (Myh6-Cre^±^-BDNF*^flox/flox^*) with mixed background. *BDNF^flox/flox^* mice were used as controls (WT). The One Step Mouse Genotyping Kit (PD101-01, Vazyme) was applied to confirm the genotypes of MYH6-Cre-BDNF^–/–^ and wild-type (WT) mice using whole genomic DNA. The following primers were used for genotyping: MYH6-Cre-F: 5′- ATG ACA GAC AGA TCC CTC CTA TCT CC-3′, MYH6-Cre-R: 5′-CTC ATC ACT CGT TGC ATC ATC GAC-3′, MYH6-Cre-F2: 5′-CAA ATG TTG CTT GTC TGG TG-3′, MYH6-Cre-R2: 5′-GTC AGT CGA GTG CAC AGT TT-3′; *BDNF^flox/flox^*-F: 5′-TGTGATTGTGTTTCTGGTGAC-3′; and *BDNF^flox/flox^*-R: 5′- GCCTTCATGCAACCGAAGTATG-3′. The MYH6-Cre-F/R primers identify the B6.FVB-Tg (Myh6-cre) allele (∼300 bp). The MYH6-Cre-BDNF-F/R primers identify the BDNF null allele (487 bp). The *BDNF^flox/flox^*-F/R primers identify the wild-type and flox alleles (∼437 bp). Animal care, surgery and handling procedures in this study were approved by the Jinan University Animal Care Committee (No. 20160413104350).

### Cardiomyocyte isolation

The cardiomyocytes of 3-month-old male MYH6-Cre-BDNF^–/–^ and WT (*BDNF^flox/flox^* mice) mice were isolated by the Langendorff method. The mice were injected with 100 μL heparin (125 U/mouse) via orbital injection. After 15 min, the abdominal cavity of the mouse was injected with 200 μL of 20% ethylurethane (40 mg/mouse), and then the cardiomyocytes were isolated by the Langendorff perfusion method. Briefly, after disinfecting the mice with 70% alcohol, the heart was quickly removed in a sterile hood and put into precooled perfusion buffer (135 mM NaCl; 14.7 mM KCl; 1 mM MgCl_2_; 10 mM HEPES; 0.33 mM NaH_2_PO_4_; 30 mM BDM; 10 mM D-glucose; 30 mM taurine; 5 mM creatine; 2 mM sodium pyruvate; 25 mM NaHCO_3_; 2 mM L-glutamine; 0.6 mM KH_2_PO_4_; 5 mM adenosine). After removing the non-cardiac tissues, the aorta of the isolated heart was inserted into the Langendorff perfusion system, fixed with fine wire, perfused at a velocity of 3 mL/min, and instilled for 3 min with perfusion buffer. Then, the heart was digested for 5–10 min with digestion buffer (0.01% collagenase II + 0.01% collagenase IV). Digestion was considered sufficient when the myocardial tissue became loose, soft and pink. The isolated heart was put into termination buffer and torn into small pieces, followed by trituration with a pipette to isolate single cardiomyocytes. After isolation, the cardiomyocyte suspension was passed through a 250 μm filter to remove the undigested tissue, and then gradient centrifugation (50 × g, 3 min; 30 × g, 2 min; 20 × g, 2 min) was used to remove the cell fragments, blood cells and endothelial cells. The isolated cardiomyocytes were suspended in termination buffer and prepared for nuclear staining and analysis. Three mice from each group were used in this experiment.

### Semiquantitative analysis of the area of cardiomyocytes and the area and number of cardiomyocyte nuclei

The cardiomyocytes of 3-month-old MYH6-Cre-BDNF^–/–^ and WT hearts were isolated by the Langendorff method as described above. Hoechst 33342 (2 μL; 5 mg/mL; Thermo, United States) was added to the cardiomyocyte suspension (1 mL; 2.5 × 10^4^ cells) and incubated at room temperature for 15 min. After three washes with termination buffer, the suspension was placed on an inverted fluorescence microscope, and images were captured with excitation at 346 nm. Image-Pro Plus 6 was used to measure the areas of cardiomyocytes and nuclei. A total of 1,007 cardiomyocytes and their respective nuclei collected from 3 male mice were analyzed in this experiment.

### Histological analysis

Hematoxylin-eosin (H&E) staining: Whole hearts were collected and fixed overnight in 4% paraformaldehyde then dehydrated, cleared, and embedded in paraffin wax. Sections (5 μm) were prepared for staining. The sections were deparaffinized in xylene (3 × 5 min) and rehydrated with successive 3-min washes in 100, 90, 80, and 70% ethanol with one final wash in tap water. The sections were then stained with hematoxylin for 5 min, rinsed with tap water for 1 min, rinsed with 1% hydrochloric acid in 80% ethanol for 5 s, rinsed with a 1% ammonia solution for 5 s, and rinsed with tap water for 1 min. The sections were then stained with eosin for 5 min and rinsed with tap water for 1 min. After dehydration in an ethanol gradient and clearing with xylene, the slides were mounted with resinene (Shanghai Yiyang Instrument Co., Ltd.). The stained sections were scanned by PANNORAMIC MIDI II (Hungary, 3DHISTECH) for image capture. The size of the ventricular chamber was measured using ImageJ software. Three mice per group were used in this experiment. The semi-quantitative analysis for cardiomyocyte death and cardiac degenerative changes (focal loss of nuclei in cardiomyocytes, muscle fiber atrophy and disorder, unclear transverse striation of myocardial fibers and interstitial loosening) is applied as follows: −: non-detectable cardiomyocyte death and cardiac degenerative change; + : scattered cardiomyocyte death and cardiac degenerative change; + + : cardiomyocyte death and cardiac degenerative change in multiple places; + + + : extensive cardiomyocyte death and cardiac degenerative change.

### Masson’s trichrome staining

Paraffin sections were dewaxed and rinsed with water using a routine protocol for H&E staining. After iron hematoxylin staining (5 min), the sections were rinsed with distilled water, differentiated with 80% ethanol containing 1% hydrochloric acid (5 s) followed by 1% ammonia solution (5 s) and rinsed with running water (2 min). Then, the sections were stained with ponceau acid fuchsin (10 min) and rinsed with distilled water. After differentiation using a phosphomolybdic acid solution (8 min), the sections were sequentially stained with aniline blue (3 min) and 1% acetic acid (2 min). After staining, the sections were dehydrated, cleared in xylene, and then mounted with resinene (Shanghai Yiyang Instrument Co., Ltd.). The stained sections were scanned by 3DHISTECH (Hungary) for image capture. The infarct size was measured using ImageJ software. Three mice per group were used in this experiment. The semi-quantitative analysis for cardiac fibrosis is used as follows: −: non-detectable fibrosis; + : scattered fibrosis; + + : extensive fibrosis.

### Wheat germ agglutinin staining

Paraffin sections were dewaxed and rinsed with water using a routine protocol for H&E staining. WGA working solution (10 μg/mL; Cat. No. W11261; Invitrogen) was prepared with distilled water, and then an appropriate amount of working solution was added to each tissue sample, and the samples were incubated at room temperature for 30 min. After two rinses with distilled water, the sections were sealed with anti-fluorescence quenching sealant. Ten random fields were captured from each slide under fluorescence microscopy (20X; Leica, Germany) at a wavelength of 488 nm. The area of cardiomyocytes was measured using Image-Pro Plus 6.0 software. Three mice per group were used in this experiment.

### Isolation of total ribonucleic acid and real-time RT-PCR

Total ribonucleic acid (RNA) was extracted using TRIzol reagent (Cat. No. 15596018; Invitrogen) according to the manufacturer’s protocol. Total RNA concentration was determined using a NanoDrop spectrophotometer (NanoDrop, Thermo Scientific). For the analysis of BDNF, ANP, BNP, α-SMA, and β-MHC gene expression, the extracted RNA (1 μg) was reverse-transcribed into first-strand cDNA using ReverTra Ace q-PCR RT Master Mix with gDNA Remover (Cat. No. FSQ-301, Toyobo) according to the manufacturer’s instructions, and then the mRNA levels were quantified using SYBR Green-based real-time PCR. The reaction mixture was composed of 10 μL of SYBR Green PCR Master Mix (Cat. No. B21202; Biotool), 0.8 μl of each primer, 6.4 μL of PCR-grade water and 2 μL of the cDNA template. The following primers were used (5′–3′): BDNF forward: TACCTGGATGCCGCAAACAT, reverse: TTTATCTGC CGCTGTGACCC; ANP forward: AGGCAGTC GATTCTGCTTGA, reverse: CGT GATAGATGAAGGCAGG AAG; BNP forward: CAGGCGGTGCTGTCTCTCTAT, reverse: GGCAGGGCATAACCCTCATA; α-SMA forward: TATCGATGACCT GGAGCTGA, reverse: AGTATTGACCTT GTCTTCCTC; β-MHC forward: GCT GTAACGCACTGAAGT TGT, reverse: TCAAAGGTGGTCCCAGAGCT; β-actin forward: CGTAAAGACCTCTATGCCAACAC, reverse: CTT GATCTTCATGGT GCTAGGAG; and GAPDH forward: TG TGTCCGTCGTGGATCTGA, reverse: CCTGCTTCACCACCT TCTTGA. Amplifications were performed with a Mini-Opticon System using the following program: 95°C for 10 min followed by 40 cycles of denaturation at 95°C for 15 s and primer annealing at 62°C for 1 min and then extension at 72°C for 30 s. Relative expression was determined using the 2^–ΔΔ*Ct*^ comparative threshold method. All cDNA samples were amplified in triplicate and were normalized to β-actin or GAPDH on the same plate. The results of this experiment were generated from 3 to 4 mice.

## Western blotting

The protein expression level of BDNF was measured by western blotting, and protein lysates of isolated cardiomyocytes were prepared in RIPA buffer (Beyotime, China) with 1 mM PMSF and a protease inhibitor cocktail (Halt Inhibitor Cocktail, Thermo, United States; Halt Phosphatase Inhibitor Cocktail, Thermo, United States). The total protein concentration was determined using a NanoDrop spectrophotometer (NanoDrop, Thermo Scientific). The protein (50 μg) was denatured in loading buffer (5 × SDS-PAGE loading buffer containing 0.5 M Tris-HCl, pH 6.8; 0.05% β-mercaptoethanol; 1% SDS; 0.005% bromophenol blue; and 50% glycerol) for 8 min at 95°C and loaded into a 15% SDS-PAGE gel. After electrophoresis, the isolated proteins were transferred onto a PVDF membrane (Bio-Rad, United States). The membrane was incubated overnight at 4°C with rabbit anti-BDNF (1:2,000, Abcam, United States) and mouse anti-GAPDH antibodies (1:4,000, Proteintech, United States) and then with donkey anti-rabbit IgG (1:5,000, Thermo, United States) and donkey anti-mouse IgG (1:5,000, Thermo, United States) secondary antibodies conjugated to horseradish peroxidase. The immunoreactive bands were detected using an ECL kit (Millipore, United States) and were analyzed using GeneSnap (Sygene). Three mice per group were used in this experiment.

### Myocardial infarction studies

MI was generated via ligation of the left anterior descending coronary artery (LAD) in 5-month-old male MYH6-Cre-BDNF^–/–^ mice and WT mice as previously described (19, 28, 29). Briefly, the mice were anesthetized with ketamine (100 mg/kg, i.p.) and underwent a left intercostal thoracotomy. The LAD was identified and then ligated directly below the left atrial appendage with 8-0-gauge nylon sutures. The presence of pallor and abnormal movement of the left ventricle (LV) confirmed LAD occlusion. The chest wall was then closed, the lungs were inflated, the mouse was extubated, and the thoracotomy was closed. After recovery, the mice were returned to the animal facility. After the cardiac function of ligated mice was analyzed 2 weeks after MI, the mice were euthanized, and their hearts were harvested. The collected hearts were fixed with 4% paraformaldehyde, embedded in paraffin wax and sectioned for further experiments. Six to eight mice were included in each group.

### Echocardiography

Transthoracic echocardiogram was performed to analyze cardiac function. In this study, 3-month-old MYH6-Cre-BDNF^–/–^ mice (male and female), 3-month-old WT mice (male and female), 8-month-old MYH6-Cre-BDNF^–/–^ mice (female), 8-month-old WT mice (female), 5-month-old MYH6-Cre-BDNF^–/–^ MI mice (male) and 5-month-old WT mice (male) were analyzed. Briefly, the mice from the above groups were anesthetized with ketamine (100 mg/kg, i.p.). The echocardiographic parameters were collected using an Acuson Sequoia 256c ultrasound system equipped with a 13-MHz linear transducer from a Vevo 770 echocardiogram (VisualSonics, Canada). The anterior chest wall was shaved, and the mouse was placed in a left lateral decubitus position. A rectal temperature probe was inserted, and the body temperature was carefully maintained between 37^°^C° and 37.5°C on a heating pad throughout the study. Parasternal long-axis, parasternal short-axis and 2 apical four-chamber views were collected in 2D-M-mode. The systolic and diastolic anatomic parameters were obtained from M-mode tracings at the mid-papillary level. The ejection fraction (EF) was calculated using the area-length method (28, 29). Three mice per group were used in this analysis.

### Transmission electron microscopy analysis

Cross-sections of samples from 8-month-old female MYH6-Cre-BDNF^–/–^ and WT hearts were fixed in a solution of 1% osmium tetroxide and 1.25% potassium ferrocyanide for 30 min at room temperature. After washing in PBS (pH 7.2) for 5 min at room temperature, the specimens were immersed overnight in 0.1% osmium tetroxide in PBS at room temperature and then processed for TEM observation. Three mice per group were used in this analysis.

### Enzyme-linked immunosorbent assay

Enzyme-linked immunosorbent assay (ELISA) kits (CUSABIO, Wuhan) were applied to analyze plasma levels of NT-proBNP, BNP, Galectin-3, BDNF, Insulin and Leptin. The detailed protocols are described in the manufacturer’s recommendations. All the parameters were analyzed in duplicate. Four to eleven mice per group were used in these assays.

### Transcriptome sequencing

Total RNA was isolated using RNeasy mini kit (Qiagen, Germany). Paired-end libraries were synthesized by using the TruSeq RNA Sample Preparation Kit (Illumina, United States) following TruSeq RNA Sample Preparation Guide. Briefly, the poly-A containing mRNA molecules were purified using poly-T oligo-attached magnetic beads. Following purification, the mRNA was fragmented into small pieces using divalent cations under 94°C for 8 min. The cleaved RNA fragments were copied into first strand cDNA using reverse transcriptase and random primers. This was followed by second strand cDNA synthesis using DNA Polymerase I and RNase H. These cDNA fragments then went through an end repair process which was added a single “A” base, and then was ligated of the adapters. The products were then purified and enriched with PCR to create the final cDNA library. Purified libraries were quantified by Qubit 2.0 Fluorometer (Life Technologies, United States) and validated by Agilent 2100 bioanalyzer (Agilent Technologies, United States) to confirm the insert size and calculate the mole concentration. Cluster was generated by cBot with the library diluted to 10 pM and then were sequenced on the Illumina HiSeq 2500 (Illumina, United States). The library construction and sequencing was performed at Shanghai Biotechnology Corporation, China.

### Ingenuity pathway analysis

The selected genes with the fold change greater than 1.5 and *Q* < 0.5 were applied to a further analysis to identify the regulatory networks and disease-function analysis using an ingenuity pathway analysis (IPA).^1^ The analysis results were applied to core-analysis in the IPA system. In the present study, only the analyzed data that were to predicted “decrease” or “increase,” which was indicated by the *Z*-score larger than 2 or less than −2, were selected as positive predictors.

### Statistics

All measured data are presented as the means ± standard errors. Two-tailed Student’s *t*-test was used to calculate the statistical significance between two groups. *P* < 0.05 was considered statistically significant.

## Results

### The one-year survival rate of MYH6-Cre-BDNF^–/–^ mice is significantly reduced

**FIGURE 1:**
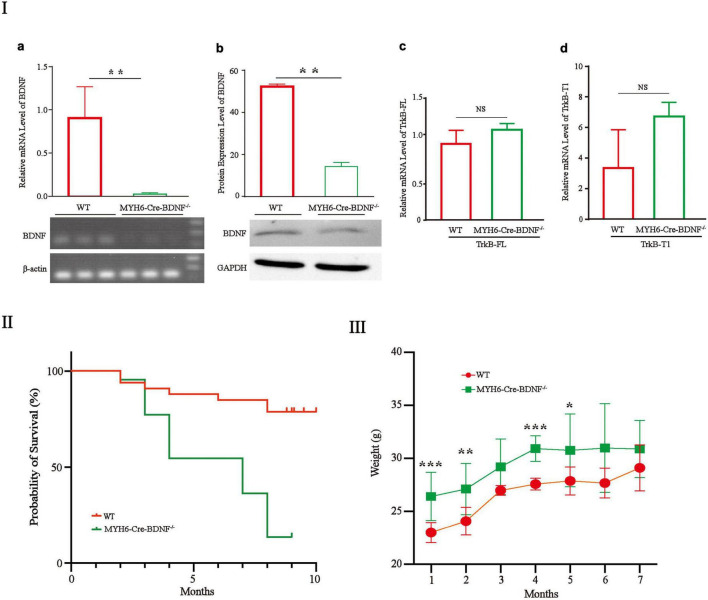
The one-year survival rate of MYH6-Cre-BDNF^–/–^ mice is significantly reduced: **(I)** The expression of BDNF at the gene **(a)** and protein levels **(b)** in MYH6-Cre-BDNF^–/–^ cardiomyocytes was significantly lower than that in WT cardiomyocytes from littermates. ^**^p < 0.01, *n* = 3. In addition, the expression of the BDNF receptors TrkB-FL and TrkB-T1 in MYH6-Cre-BDNF^–/–^ cardiomyocytes was not significantly different from that in WT cardiomyocytes **(c,d)**. **(II)** The one-year survival rate of MYH6-Cre-BDNF^–/–^ mice was significantly lower than that of WT mice, *p* < 0.01. **(III)** The body weights of MYH6-Cre-BDNF^–/–^ mice in 1-, 2-, 4-, and 5-month-old of age were significantly higher than those of WT mice. *p < 0.05, **p < 0.01, ***p < 0.001, *n* = 12 for the 1-month-old WT and MYH6-Cre-BDNF^–/–^ groups respectively. *n* = 10 for the 2-month-old WT and MYH6-Cre-BDNF^–/–^ groups respectively. *n* = 5 and 7 for the 3-month-old group WT and MYH6-Cre-BDNF^–/–^ groups. *n* = 10 and 3 for the 4-month-old WT and MYH6-Cre-BDNF^–/–^ groups. *n* = 8 for the 5-month-old WT and MYH6-Cre-BDNF^–/–^ groups. *n* = 3 and 6 for the 6-month-old WT and MYH6-Cre-BDNF^–/–^ groups. *n* = 3 for the 7-month-old WT and MYH6-Cre-BDNF^–/–^ groups respectively.

The gene and protein expression levels of BDNF in MYH6-Cre-BDNF^–/–^ cardiomyocytes were significantly lower than those in cardiomyocytes from WT littermates (hereafter referred to as “WT”; comparisons are made between same-sex littermates) (Figures 1Ia,b; *p* < 0.01). In addition, the expression of the BDNF receptors TrkB-FL and TrkB-T1 in MYH6-Cre-BDNF^–/–^ cardiomyocytes was not significantly different from that in WT cardiomyocytes (Figures 1Ic,d; *p* > 0.05). The embryonic development and reproduction of MYH6-Cre-BDNF^–/–^ mice did not show abnormal effects, as MYH6-Cre-BDNF^–/–^ mice were able to grow and reproduce successfully. However, the one-year survival rate of MYH6-Cre-BDNF^–/–^ mice (13.6%) was significantly lower than that of WT mice (78.8%) (Figure 1II; *p* < 0.01). In addition, the body weights of MYH6-Cre-BDNF^–/–^ mice observed from 2 to 7 months of age were significantly higher than those of WT mice (Figure 1III; *p* < 0.01).

### Cardiac dysfunction and somatic edema are found in adult MYH6-Cre-BDNF^–/–^ mice

**FIGURE 2:**
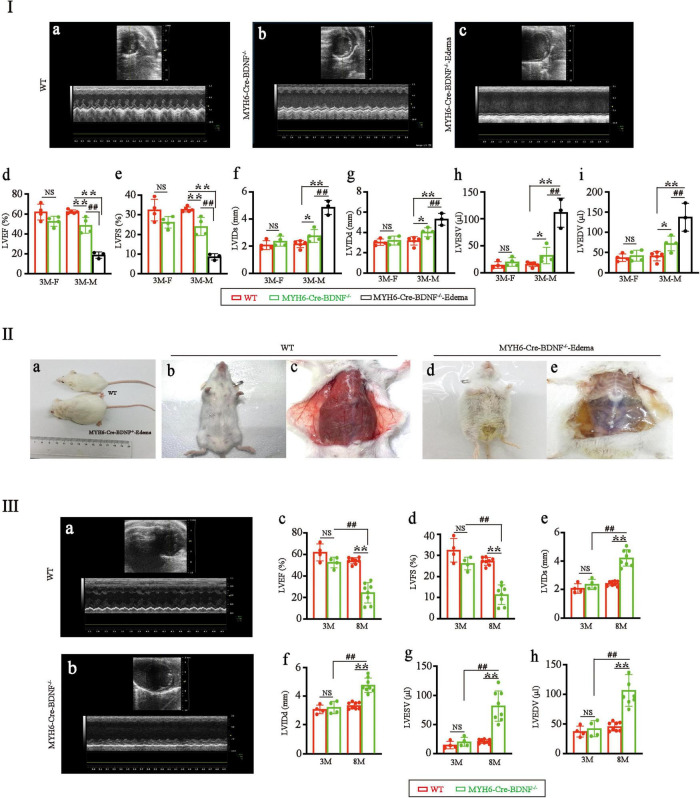
Cardiac dysfunction and osmotic edema are found in adult MYH6-Cre-BDNF^–/–^ mice: **(I)** Representative echocardiography of WT hearts **(a)**, MYH6-Cre-BDNF^–/–^ hearts **(b)** and MYH6-Cre-BDNF^–/–^-edema hearts **(c)**. Echocardiography showed that in 3-month-old males, the left ventricular ejection fraction (LVEF) **(d)** and left ventricular fractional shortening (LVFS) **(e)** of MYH6-Cre-BDNF^–/–^ hearts were significantly lower than those of WT hearts, while the left ventricular end-systolic internal diameter (LVIDs) **(f)**, left ventricular end-diastolic internal diameter (LVIDd) **(g)**, left ventricular end-systolic volume (LVESV) **(h)** and left ventricular end-diastolic volume (LVEDV) **(i)** of MYH6-Cre-BDNF^–/–^ hearts were significantly higher than those of WT hearts. Three-month-old osmotic edema MYH6-Cre-BDNF^–/–^ male mice had worse cardiac function parameters (LVEF, LVFS, LVIDs, LVIDd, LVESV and LVEDV) than 3-month-old WT mice **(a–i)**. *p < 0.05, **, ^##^*p* < 0.01. *n* = 4 for the 3-month-old female WT and MYH6-Cre-BDNF^–/–^ groups respectively; *n* = 5 and 4 for the 3-month-old male WT and MYH6-Cre-BDNF^–/–^ groups. *n* = 3 for the 3-month-old male MYH6-Cre-BDNF^–/–^ edema group. **(II)** Starting at 3 months of age, some MYH6-Cre-BDNF^–/–^ mice exhibited osmotic edema **(a,d,e)** compared to WT mice **(a–c)**. **(III)** Representative echocardiography of 8-month-old WT hearts **(a)** and 8-month-old MYH6-Cre-BDNF^–/–^ hearts **(b)**. The LVEF **(c)** and LVFS **(d)** of 8-month-old MYH6-Cre-BDNF^–/–^ female hearts were significantly smaller than those of 8-month-old WT female hearts, 3-month-old MYH6-Cre-BDNF^–/–^ female hearts and 3-month-old WT female hearts, while LVIDs **(e)**, LVIDd **(f)**, LVESV **(g)**, and LVEDV **(h)** of 8-month-old MYH6-Cre-BDNF^–/–^ female hearts were significantly higher than those of 8-month-old WT female hearts, 3-month-old MYH6-Cre-BDNF^–/–^ female hearts and 3-month-old WT female hearts. **, ^##^*p* < 0.01. *n* = 4 for the 3-month-old female WT and MYH6-Cre-BDNF^–/–^ groups respectively; *n* = 8 for the 8-month-old female WT and MYH6-Cre-BDNF^–/–^ groups respectively.

Echocardiography documented that in 3-month-old males, the left ventricular ejection fraction (LVEF) and left ventricular fractional shortening (LVFS) of MYH6-Cre-BDNF^–/–^ hearts were significantly lower than those of WT hearts (Figures 2Ia,b,d,e; *p* < 0.05), while the left ventricular end-systolic internal diameter (LVIDs), left ventricular end-diastolic internal diameter (LVIDd), left ventricular end-systolic volume (LVESV) and left ventricular end-diastolic volume (LVEDV) of MYH6-Cre-BDNF^–/–^ hearts were significantly higher than those of WT hearts (Figure 2Ia,b,f–i; *p* < 0.05). From 3 months of age onwards, some MYH6-Cre-BDNF^–/–^ mice had osmotic edema, which had been never occurred in WT mice (21 osmotic edema cases [male: 9; female: 12] out of 55 MYH6-Cre-BDNF^–/–^ mice; approximately 38.2%; edema ratio: 40% for male, 36% for female), and 3-month-old male MYH6-Cre-BDNF^–/–^ mice with osmotic edema had worse cardiac function parameters (LVEF, LVFS, LVIDs, LVIDd, LVESV and LVEDV) than 3-month-old WT mice (Figure 2Ia–i; *P* < 0.05). All MYH6-Cre-BDNF^–/–^ mice died approximately 1 week after somatic edema occurred (Figure 2IIa–e).

In addition, all the observed parameters tended to be worse in 3-month-old MYH6-Cre-BDNF^–/–^ female mice than in 3-month-old female WT mice of the same litter; however, the differences were not statistically significant (Figure 2Ia–i; *P* > 0.05). To investigate whether cardiac dysfunction also occurs in middle-aged female MYH6-Cre-BDNF^–/–^ mice, 8-month-old MYH6-Cre-BDNF^–/–^ female mice were observed. The LVEF and LVFS of 8-month-old MYH6-Cre-BDNF^–/–^ female hearts were significantly smaller than those of 8-month-old WT female hearts, and the same result was observed at 3 months of age (Figures 2IIIa–d; *P* < 0.01), while the LVIDs, LVIDd, LVESV, and LVEDV of 8-month-old MYH6-Cre-BDNF^–/–^ hearts were significantly higher than those of 8- and 3-month-old WT hearts as well as 3-month-old MYH6-Cre-BDNF^–/–^ hearts (Figures 2IIIa,b,e–h; *P* < 0.05). These results suggested that the pathological phenotypes that occurred in MYH6-Cre-BDNF^–/–^ mice were not sex dependent; however, the time window of cardiac dysfunction in female MYH6-Cre-BDNF^–/–^ hearts was later than that in male MYH6-Cre-BDNF^–/–^ hearts. Therefore, the subsequent investigation of other parameters focused on male mice.

### Cardiomyocyte hypertrophy, increased areas of cardiomyocyte cells and nuclei, and an increased percentage of single-nucleus cardiomyocytes are found in young adult MYH6-Cre-BDNF^–/–^ mice

**FIGURE 3:**
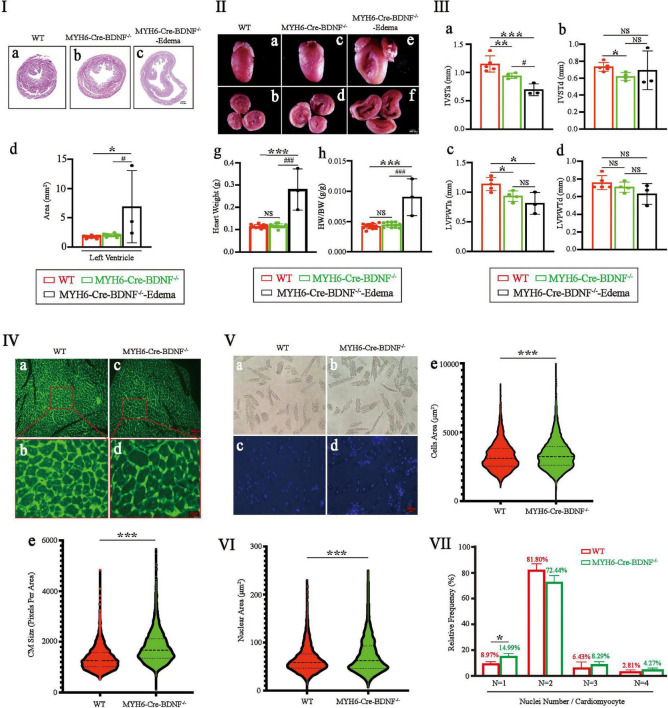
Eccentric myocardial hypertrophy accompanied by an increase in the area of the cardiomyocyte nucleus and an increase in the percentage of single-nucleus cardiomyocytes were found in young adult MYH6-Cre-BDNF^–/–^ mice: **(I,II)** The left ventricular area of 3-month-old MYH6-Cre-BDNF^–/–^ male hearts was significantly larger than that of WT hearts **(I)**. In addition, the ventricular area, heart weight and ratio of heart weight/body weight of 3-month-old MYH6-Cre-BDNF^–/–^ osmotic edema mice were significantly higher than those of 3-month-old MYH6-Cre-BDNF^–/–^ and WT mice **(I,II)**. *n* = 10 for the 3-month-old male WT and MYH6-Cre-BDNF^–/–^ groups respectively; *n* = 3 for the 3-month-old male MYH6-Cre-BDNF^–/–^ edema group. **(III)** Echocardiography showed that the interventricular septum end-systolic thickness (IVSTs) and left ventricular posterior wall end-systolic thickness (LVPWTs) of 3-month-old MYH6-Cre-BDNF^–/–^ hearts were significantly smaller than those of WT hearts **(a,c)**. In addition, the IVSTs of 3-month-old MYH6-Cre-BDNF^–/–^ osmotic edema hearts was significantly smaller than those of 3-month-old MYH6-Cre-BDNF^–/–^ hearts and WT hearts **(c)**. Furthermore, the interventricular septum end-diastolic thickness (IVSTd) of 3-month-old MYH6-Cre-BDNF^–/–^ hearts was significantly smaller than that of WT hearts **(b)**. *n* = 5 and 4 for the 3-month-old male WT and MYH6-Cre-BDNF^–/–^ groups respectively; *n* = 3 for the 3-month-old male MYH6-Cre-BDNF^–/–^ edema group. **(IV)** Semiquantification of WGA immunofluorescence staining revealed that the mean area of cardiomyocytes in 3-month-old MYH6-Cre-BDNF^–/–^ hearts was significantly higher than that in WT hearts. *n* = 6. **(V)** Quantification analysis of isolated single cardiomyocytes demonstrated that the mean area of cardiomyocytes in 3-month-old MYH6-Cre-BDNF^–/–^ hearts was significantly higher than that in WT hearts. *n* = 3. **(VI)** The mean area of cardiomyocyte nuclei of 3-month-old MYH6-Cre-BDNF^–/–^ hearts was significantly higher than that of WT hearts. *n* = 3. **(VII)** The percentage of single-nucleus cardiomyocytes in 3-month-old MYH6-Cre-BDNF^–/–^ hearts (N: Number of nuclei per cell) was significantly higher than that in WT hearts, *n* = 3. *, ^#^*p* < 0.05, **p < 0.01, ***, ^###^*p* < 0.001.

The left ventricular area of 3-month-old MYH6-Cre-BDNF^–/–^ hearts was significantly larger than that of WT hearts (Figure 3I; *p* < 0.05). In contrast, the differences of heart weight and the ratio of heart weight/body weight were not statistics significance between 3-month-old MYH6-Cre-BDNF^–/–^ mice and 3-month-old WT mice (Figures 3IIg,h; *p* > 0.05). However, all three parameters of 3-month-old MYH6-Cre-BDNF^–/–^ osmotic edema mice were significantly higher than those of 3-month-old MYH6-Cre-BDNF^–/–^ and WT mice (*p* < 0.0001) (Figures 3I,II). Echocardiography documented that the interventricular septum end-systolic thickness (IVSTs) and left ventricular posterior wall end-systolic thickness (LVPWTs) of 3-month-old MYH6-Cre-BDNF^–/–^ hearts and MYH6-Cre-BDNF^–/–^ osmotic edema hearts were smaller than those of WT hearts (Figures 3IIIa,c; *P* < 0.05). Furthermore, the interventricular septum end-diastolic thickness (IVSTd) of 3-month-old MYH6-Cre-BDNF^–/–^ hearts was smaller than that of WT hearts (Figure 3IIIb; *P* < 0.05). The difference in left ventricular posterior wall end-diastolic thickness (LVPWTd) among WT, 3-month-old MYH6-Cre-BDNF^–/–^ hearts and 3-month-old MYH6-Cre-BDNF^–/–^ osmotic edema hearts was not statistically significant (Figure 3IIId; *P* > 0.05). In parallel, semiquantification of WGA immunofluorescence staining revealed that the mean area of cardiomyocytes in 3-month-old MYH6-Cre-BDNF^–/–^ hearts was significantly higher than that in WT hearts (Figure 3IV; *P* < 0.05). Furthermore, the quantitative analysis of isolated single cardiomyocytes demonstrated that the mean area of cardiomyocytes from 3-month-old MYH6-Cre-BDNF^–/–^ hearts was significantly higher than that of WT hearts (Figure 3V; *P* < 0.05). The findings revealed cardiomyocyte hypertrophy in 3-month-old MYH6-Cre-BDNF^–/–^ hearts, while this pathological change was more serious in 3-month-old MYH6-Cre-BDNF^–/–^ osmotic edema hearts. In addition, the mean area of cardiomyocyte nuclei in 3-month-old MYH6-Cre-BDNF^–/–^ hearts was significantly higher than that in WT hearts (Figure 3VI; *P* < 0.05). The percentage of single-nucleus cardiomyocytes in 3-month-old MYH6-Cre-BDNF^–/–^ hearts was significantly higher than that in WT hearts (Figure 3VII; *P* < 0.05).

### Cardiomyocyte death, degenerative changes in the myocardium (degeneration of cardiomyocytes, increased interstitial fibrosis, serious mitophagy and swelling of mitochondria) and left atrial appendage thrombus are found in young adult MYH6-Cre-BDNF^–/–^ hearts

**FIGURE 4:**
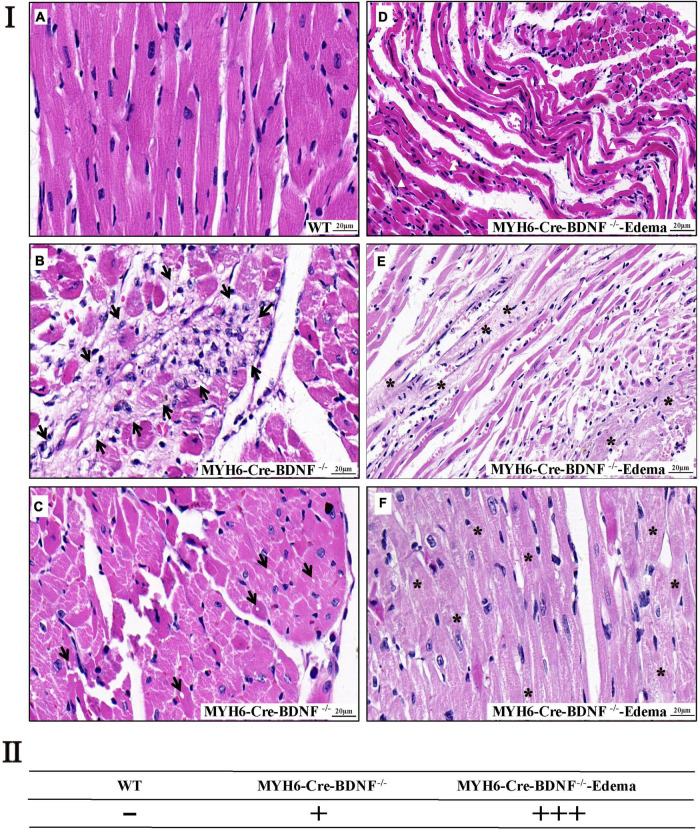
Cardiomyocyte death and degenerative changes in the myocardium are found in young adult MYH6-Cre-BDNF^–/–^ hearts **(I)** H&E histological staining revealed that in 3-month-old MYH6-Cre-BDNF^–/–^ hearts but not in WT hearts **(a)**, focal cardiomyocyte death, focal degenerative changes in cardiomyocytes, focal inflammation in the myocardium (**b**; arrow) and focal loss of nuclei in cardiomyocytes (**c**; arrow) were found, while in 3-month-old osmotic edema MYH6-Cre-BDNF^–/–^ hearts, in addition to the above pathological changes, severe muscle fiber atrophy and disorder (**d,e**; white triangle), unclear transverse striation of myocardial fibers (**e,f**; asterisk) and interstitial loosening **(d,e)** were also found in the myocardium. *n* = 5 and 4 for the 3-month-old male WT and MYH6-Cre-BDNF^–/–^ groups respectively; *n* = 3 for the 3-month-old male MYH6-Cre-BDNF^–/–^ edema group. **(II)** Semi-quatitation of I. *n* = 3. Bar size as shown in the figure.

In addition to cardiomyocyte hypertrophy, H&E histological staining revealed that, focal cardiomyocyte death, focal degenerative changes in cardiomyocytes, focal loss of nuclei in cardiomyocytes and focal inflammation in the myocardium were found in 3-month-old MYH6-Cre-BDNF^–/–^ hearts but not in WT hearts (Figures 4Ia–c,II). In 3-month-old MYH6-Cre-BDNF^–/–^ hearts with osmotic edema, in addition to the above pathological changes, severe muscle fiber atrophy and disorder, unclear transverse striation of myocardial fibers and interstitial loosening were also found in the myocardium (Figures 4Id–f,II). Moreover, left atrial appendage thrombosis was found in most osmotic edema MYH6-Cre-BDNF^–/–^ hearts (Supplementary Figures 1A,E,H). In addition to left atrial appendage thrombosis, some MYH6-Cre-BDNF^–/–^ osmotic edema hearts also showed right atrial appendage thrombosis, left and right atrial thrombosis and left ventricular thrombosis (Supplementary Figures 1E–K). In addition, H&E staining revealed that the thrombosis had a mixed pathological morphology that was infiltrated with red blood cells, macrophages, lymphocytes and nodular eosinophilic material (Supplementary Figures 1F,H–K). Degenerative changes in the myocardium occurred in MYH6-Cre-BDNF^–/–^ hearts, leading us to further observe cardiac fibrosis. Masson’s trichrome staining revealed that compared to WT myocardium, MYH6-Cre-BDNF^–/–^ myocardium had more extensive perivascular fibrosis and scattered focal interstitial fibrosis, mainly in the inner myocardium (Supplementary Figure 2). Furthermore, in MYH6-Cre-BDNF^–/–^ osmotic edema hearts, more extensive interstitial fibrosis was found in the myocardium and thromboses, which were located in the atrial appendage and atrial and ventricular cavity, than in MYH6-Cre-BDNF^–/–^ hearts and WT hearts (Supplementary Figure 3). Moreover, more extensive cardiomyocyte death and interstitial fibrosis were observed in 8-month-old MYH6-Cre-BDNF^–/–^ osmotic edema myocardium than in 8-month-old MYH6-Cre-BDNF^–/–^, 8-month-old WT, 3-month-old MYH6-Cre-BDNF^–/–^ osmotic edema and 3-month-old MYH6-Cre-BDNF^–/–^ myocardium (Supplementary Figure 4 vs. Figure 4 and Supplementary Figure 2). In addition, the above pathological phenotypes were more severe in 8-month-old WT myocardium than in 3-month-old WT myocardium (Supplementary Figure 4 vs. Figure 4 and Supplementary Figure 2). In addition, in 8-month-old MYH6-Cre-BDNF^–/–^ myocardium, focal interstitial fibrosis was found between the inner and external myocardium (Supplementary Figure 4); in contrast, the focal interstitial fibrosis found in 3-month-old MYH6-Cre-BDNF^–/–^ myocardium was mainly located in the inner myocardium (Supplementary Figures 2E,F). Furthermore, transmission electron microscopy (TEM) revealed significantly disrupted mitochondrial distribution, swollen mitochondria, decreased mitochondrial density and serious mitophagy as well as blurred myofilament structures in cardiomyocytes, unclear sarcomere structures and Z lines, and unclear boundaries between the dark zone and the bright zone in 8-month-old MYH6-Cre-BDNF^–/–^ hearts (Supplementary Figure 5).

### Significant increases in heart failure indices, cardiomyocyte hypertrophy, and increased serum brain-derived neurotrophic factor and insulin levels are found in young adult MYH6-Cre-BDNF^–/–^ mice

**FIGURE 5:**
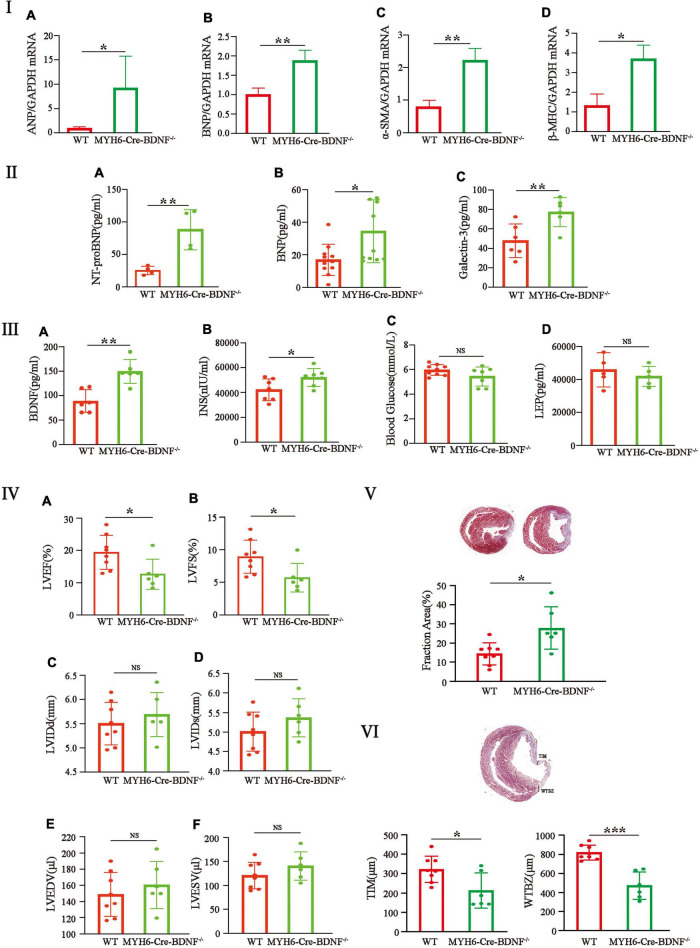
Significant increases in heart failure indices, pathological hypertrophy in the left ventricle, serum BDNF and insulin levels and poor cardiac function and regeneration are found in young adult MYH6-Cre-BDNF^–/–^ mice: **(I–III)** The qPCR results showed that the expression of the heart failure markers atrial natriuretic peptide (ANP) **(Ia)** and brain natriuretic peptide (BNP) **(Ib)** was significantly higher in the left ventricle of 3-month-old MYH6-Cre-BDNF^–/–^ hearts than in that of WT hearts. The analysis of the heart failure indices, N-terminal pro-brain natriuretic peptide (NT-proBNP), BNP and Galectin-3, also confirmed that serum levels of NT-proBNP **(IIa)**, BNP **(IIb)** and Galectin-3 **(IIc)** in 3-month-old MYH6-Cre-BDNF^–/–^ mice were significantly higher than those in WT mice. In addition, qPCR showed that compared to WT, the expression of the myocardial hypertrophy markers alpha smooth muscle actin (αSMA) **(Ic)** and beta myosin heavy chain (β-MHC) **(Id)** was significantly higher in the left ventricle of 3-month-old MYH6-Cre-BDNF^–/–^ mice. Moreover, compared to those in WT mice, the metabolism-related parameters, serum levels of BDNF and insulin in 3-month-old MYH6-Cre-BDNF^–/–^ mice were significantly increased **(IIIa,b)**. *n* = 3 for the WT and MYH6-Cre-BDNF^–/–^ groups respectively in **(Ia,b)**; *n* = 4 for the WT and MYH6-Cre-BDNF^–/–^ groups respectively in **(IIa)**; *n* = 11 for the WT and MYH6-Cre-BDNF^–/–^ groups respectively in **(IIb)**; *n* = 6 for the WT and MYH6-Cre-BDNF^–/–^ groups respectively in **(IIc)**; *n* = 6 for the WT and MYH6-Cre-BDNF^–/–^ groups respectively in **(IIIa)**; *n* = 7 for the WT and MYH6-Cre-BDNF^–/–^ groups respectively in **(IIIb)**; *n* = 9 and 7 for the WT and MYH6-Cre-BDNF^–/–^ groups respectively in **(IIIc)**; *n* = 5 for the WT and MYH6-Cre-BDNF^–/–^ groups respectively in **(IIId)**. **(IV–VI)** After MI, the LVEF and LVFS of 5-month-old MYH6-Cre-BDNF^–/–^ mice were significantly smaller than those of WT mice **(IVa,b)**. While the LVIDd, LVIDs, LVEDV, and LVESV were not statistically significant compared to those in WT mice **(IVc–f)**. In parallel, the infarct size of MYH6-Cre-BDNF^–/–^ MI hearts were significantly larger than that of WT hearts **(V)**. In addition, the smallest ventricular wall thickness of the infarct zone and the thickness of the infarct border zone of MYH6-Cre-BDNF^–/–^ MI hearts were significantly smaller than those of wild-type MI hearts **(VI)**. *n* = 8 and 6 for the WT and MYH6-Cre-BDNF^–/–^ groups respectively in **(IV–VI)**. LVEF, Left ventricular ejection fraction; LVFS, Left ventricular fractional shortening; LVIDs, Left ventricular end-systolic internal diameter; LVIDd, Left ventricular end-diastolic internal diameter; LVESV, Left ventricular end-systolic volume; LVEDV, Left ventricular end-diastolic volume; MYH6-Cre-BDNF^–/–^, Cardiomyocyte-derived BDNF conditional knockout. *p < 0.05, **p < 0.01, ***p < 0.001.

The HF phenotypes (such as osmotic edema, cardiomyocyte hypertrophy, increased cardiomyocyte death, degeneration of the myocardium and cardiac dysfunction) that occurred in MYH6-Cre-BDNF^–/–^ mice led us to investigate HF parameters in the myocardium and peripheral serum. The qPCR results showed that, 3-month-old MYH6-Cre-BDNF^–/–^ hearts showed significantly higher expression of the HF markers atrial natriuretic peptide (ANP) and brain natriuretic peptide (BNP) in the left ventricle than WT hearts (Figures 5Ia,b; *p* < 0.05). Analysis of the HF indices N-terminal pro-brain natriuretic peptide (NT-proBNP), BNP and Galectin-3 also confirmed that serum levels of NT-proBNP, BNP and Galectin-3 in 3-month-old MYH6-Cre-BDNF^–/–^ mice were significantly higher than those in WT mice (Figures 5IIa–c; *p* < 0.05). In addition, qPCR showed that 3-month-old MYH6-Cre-BDNF^–/–^ mice showed significantly higher expression of the cardiomyocyte hypertrophy markers alpha smooth muscle actin (αSMA) and beta myosin heavy chain (β-MHC) in the left ventricle than WT mice (Figures 5Ic,d; *p* < 0.05). These results confirm that HF and dilated cardiomyopathy occurred in 3-month-old MYH6-Cre-BDNF^–/–^ hearts. Metabolism-related parameters (glucose, insulin, leptin and BDNF) were also measured. Compared to those in WT mice, the serum levels of BDNF and insulin in 3-month-old MYH6-Cre-BDNF^–/–^ mice were significantly increased (Figures 5IIIa,b; *p* < 0.01). While the differences in glucose and leptin levels were not statistically significant between 3-month-old MYH6-Cre-BDNF^–/–^ mice and 3-month-old WT mice (Figures 5IIIc,d; *p* > 0.05). Metabolic disruption of BDNF, increased expression of non-cardiomyocyte-derived BDNF and activation of insulin metabolism were observed in 3-month-old MYH6-Cre-BDNF^–/–^ mice.

### Poor cardiac function and regeneration are found in young adult MYH6-Cre-BDNF^–/–^ hearts during myocardial infarction

The possible damaging effect on myocardial regeneration in MYH6-Cre-BDNF^–/–^ hearts during MI was also investigated. The one-year survival rate of MYH6-Cre-BDNF^–/–^ mice showed that the survival rate of 4–6-month-old mice decreased to approximately 58% from approximately 80% in 3-month-old mice; therefore, 5-month-old male MYH6-Cre-BDNF^–/–^ mice were used in this experiment. After MI, the LVEF and LVFS of MYH6-Cre-BDNF^–/–^ mice were apparently smaller (Figures 5IVa,b; *p*< 0.05), while the LVIDd, LVIDs, LVEDV and LVESV were not statistically significant compared to those in WT mice (Figures 5IVc–f; *p*> 0.05). Furthermore, the infarct size of MYH6-Cre-BDNF^–/–^ MI hearts was significantly larger than that of WT hearts (Figure 5V; *p* < 0.05). In addition, the smallest ventricular wall thickness of the infarct zone and the thickness of the infarct border zone of MYH6-Cre-BDNF^–/–^ MI hearts were significantly smaller than those of wild-type MI hearts (Figure 5VI; *p* < 0.05).

### Transcriptome sequencing combined with ingenuity pathway analysis reveals the pathways and gene interaction networks involved in cardiomyocyte death, myocardial degeneration, cardiac inflammation, ROS production, body weight increase and metabolic disruption seen in MYH6-Cre-BDNF^–/–^ mice

**FIGURE 6:**
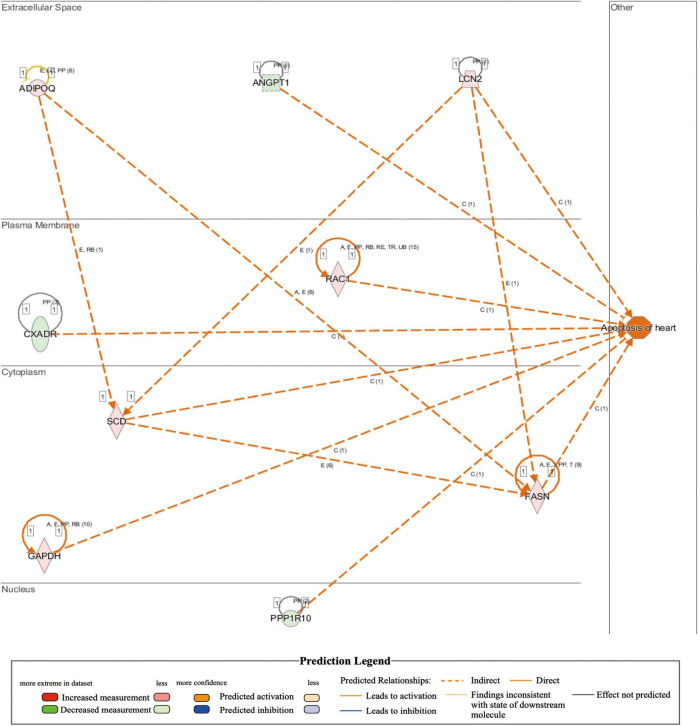
Activated regulation of heart apoptosis played a role in heart failure induced by loss of cardiomyocyte-derived BDNF: Integrated IPA identified that apoptosis in cardiomyocyte-derived BDNF conditional knockout hearts was higher than that in wild-type hearts (*p*-value = 2.06E-02; activation *z*-score = 2.028). The interaction network demonstrated that 9 genes (6 upregulated genes and 3 downregulated genes) were included that were involved in the activation of apoptosis in cardiomyocyte-derived BDNF conditional knockout hearts. Note: The shown interaction network only showed the predicted interaction by IPA analysis. Other interaction networks shown in this study are presented as the same format.

**FIGURE 7:**
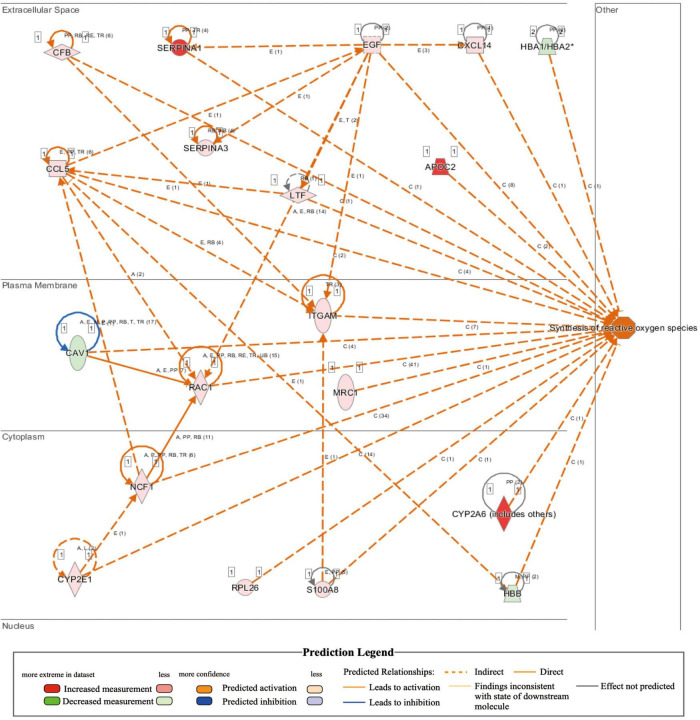
Activated regulation of reactive oxygen species synthesis played a role in heart failure induced by the loss of cardiomyocyte-derived BDNF: IPA revealed that the synthesis of reactive oxygen species in cardiomyocyte-derived BDNF conditional knockout hearts was higher than that in wild-type hearts (*p*-value = 6.05E-06; activation *z*-score = 2.418). The interaction network demonstrated that 19 genes (16 upregulated genes and 3 downregulated genes) were included, as shown in the interaction network for the activation of reactive oxygen species synthesis in cardiomyocyte-derived BDNF conditional knockout hearts.

Comparison of whole-transcriptome sequencing between 3-month-old male MYH6-Cre-BDNF^–/–^ hearts and WT hearts combined with IPA was applied to identify underlying gene interaction networks and pathways affected by the deletion of cardiomyocyte-derived BDNF. A total of 501 differentially expressed genes (fold change ≥ 1.5; *q* < 0.5) were selected for IPA to identify the genes related to canonical pathways, biofunctions and diseases, and interaction networks. Indeed, IPA revealed that the differential expression of 9 genes (6 upregulated genes and 3 downregulated genes; MYH6-Cre-BDNF^–/–^ vs. WT; all comparisons hereafter are the same) was related to increased apoptotic activity in the heart (Table 1). The interaction network of these 9 genes to regulate the activation of heart apoptosis was revealed by IPA, as shown in Figure 6, which provided a possible molecular mechanism for the cardiomyocyte death seen in MYH6-Cre-BDNF^–/–^ hearts. In addition, the synthesis of reactive oxygen species (ROS) was increased in MYH6-Cre-BDNF^–/–^ hearts compared to WT hearts, which was related to 19 genes (upregulated: 16 genes; downregulated: 3 genes; Table 2) via their interaction network to regulate the increase in ROS, as shown in Figure 7. In addition, the IPA for canonical pathways revealed that the pathway for the production of nitric oxide and reactive oxygen species in macrophages was activated in MYH6-Cre-BDNF^–/–^ hearts compared to WT hearts and was related to 7 genes (upregulated: 6 genes; downregulated: 1 gene; Table 3) via their interaction network, which regulated this effect, as shown in Supplementary Figure 6. The findings provide a molecular mechanism and the relevant genes for the cardiomyocyte death, cardiac dysfunction, cardiomyocyte degeneration and hypotrophy, HF and increased ROS activity seen in MYH6-Cre-BDNF^–/–^ hearts.

**TABLE 1:** IPA analysis identifies increase of apoptosis of heart in MYH6-Cre-BDNF^–/–^ heart (activation *z*-score = 2.028; *p* = 2.06E-02).

Genes	ID	Expr log ratio	Phospho false discovery	Location
ADIPOQ	ENSMUSG00000022878	↑1.964	1.08E-05	Extracellular space
ANGPT1	ENSMUSG00000022309	↓-0.815	3.18E-01	Extracellular space
CXADR	ENSMUSG00000022865	↓-0.598	1.08E-01	Plasma membrane
FASN	ENSMUSG00000025153	↑1.524	4.21E-03	Cytoplasm
GAPDH	ENSMUSG00000057666	↑2.240	1.23E-01	Cytoplasm
LCN2	ENSMUSG00000026822	↑1.344	4.48E-01	Extracellular space
PPP1R10	ENSMUSG00000039220	↓-0.633	1.48E-01	Nucleus
RAC1	ENSMUSG00000001847	↑1.404	6.32E-02	Plasma membrane
SCD	ENSMUSG00000037071	↑2.001	7.42E-03	Cytoplasm

**TABLE 2:** IPA analysis identifies increase of synthesis of reactive oxygen species in MYH6-Cre-BDNF^–/–^ heart (activation *z*-score = 2.418; *p* = 6.05E-06).

Genes	ID	Expr log ratio	Phospho false discovery	Location
CYP2A6	ENSMUSG00000000547	↑∞	2.00E-01	Cytoplasm
APOC2	ENSMUSG00000002992	↑∞	1.52E-01	Extracellular space
SERPINA1	ENSMUSG00000072849	↑∞	4.78E-01	Extracellular space
SERPINA3	ENSMUSG00000021091	↑1.304	4.75E-03	Extracellular space
RPL26	ENSMUSG00000060938	↑6.286	5.03E-95	Cytoplasm
LTF	ENSMUSG00000032496	↑3.135	2.34E-01	Extracellular space
CCL5	ENSMUSG00000035042	↑2.969	4.72E-01	Extracellular space
S100A8	ENSMUSG00000056054	↑2.748	1.82E-01	Cytoplasm
CYP2E1	ENSMUSG00000025479	↑1.893	1.70E-08	Cytoplasm
RAC1	ENSMUSG00000001847	↑1.404	6.32E-02	Plasma membrane
ITGAM	ENSMUSG00000030786	↑1.227	4.75E-03	Plasma membrane
CFB	ENSMUSG00000090231	↑1.044	1.31E-03	Extracellular space
EGF	ENSMUSG00000028017	↑0.834	9.48E-02	Extracellular space
CXCL14	ENSMUSG00000021508	↑0.718	3.33E-01	Extracellular space
NCF1	ENSMUSG00000015950	↑0.688	2.77E-01	Cytoplasm
MRC1	ENSMUSG00000026712	↑0.610	1.59E-03	Plasma membrane
HBB	ENSMUSG00000052305	↓-0.740	2.93E-01	Cytoplasm
CAV1	ENSMUSG00000007655	↓-0.872	1.81E-02	Plasma membrane
HBA1/HBA2	ENSMUSG00000069917	↓-0.944	4.36E-04	Extracellular space

**TABLE 3:** IPA analysis identifies activation of pathway for production of nitric oxide and reactive oxygen species in macrophages in MYH6-Cre-BDNF^–/–^ heart (activation *z*-score = 2.65; *p* = 3.51E-02).

Genes	ID	Expr log ratio	Phospho false discovery	Location
APOC2	ENSMUSG00000002992	↑∞	1.52E-01	Extracellular space
LYZ	ENSMUSG00000069515	↑1.139	4.23E-01	Extracellular space
NCP1	ENSMUSG00000015950	↑0.688	2.77E-01	Cytoplasm
PPP1R10	ENSMUSG00000039220	↓-0.633	1.48E-01	Nucleus
RAC1	ENSMUSG00000001847	↑1.404	6.32E-02	Plasma membrane
S100A8	ENSMUSG00000056054	↑2.748	1.82E-01	Cytoplasm
SERPINA1	ENSMUSG00000072849	↑∞	4.78E-01	Extracellular space

In addition, IPA identified that the regulation of weight gain was increased in differentially expressed genes between MYH6-Cre-BDNF^–/–^ mice and WT mice; (9 upregulated genes; Supplementary Table 1) and their interaction network (Supplementary Figure 7) were related to this function. Moreover, the activity of lipid metabolism was discovered to be increased in MYH6-Cre-BDNF^–/–^ mice compared to WT mice. The activated functions included synthesis of fatty acids (11 genes upregulated and 1 gene downregulated, as shown in Supplementary Table 2 and in the interaction network in Supplementary Figure 8), synthesis of lipids (15 genes upregulated and 2 genes downregulated, as shown in Supplementary Table 3 and in the interaction network in Supplementary Figure 9), biosynthesis of polyunsaturated fatty acids (8 upregulated genes and 1 downregulated gene, as shown in Supplementary Table 4 and in the interaction network in Supplementary Figure 10), synthesis of eicosanoids (8 upregulated genes and 1 downregulated gene, as shown in Supplementary Table 5 and in the interaction network in Supplementary Figure 11), the concentration of colfosceril palmitate (4 upregulated genes, as shown in Supplementary Table 6 and in the interaction network in Supplementary Figure 12) and concentration of triacylglycerol (11 genes upregulated and 2 genes downregulated, as shown in Supplementary Table 7 and in the interaction network in Supplementary Figure 13) as well as fatty acid metabolism (17 upregulated genes and 1 downregulated gene, as shown in Supplementary Table 8 and in the interaction network in Supplementary Figure 14). Furthermore, IPA of canonical pathways identified that the STAT3, melatonin degradation I and acetone degradation I (to methylglyoxal) pathways were activated in MYH6-Cre-BDNF^–/–^ hearts. The increase in the expression of 6 genes with the interaction network for STAT3 pathway activation is shown in Supplementary Table 9 and Supplementary Figure 15. The increase in the expression of 4 genes in the interaction network for the activation of melatonin degradation I and acetone degradation I is shown in Supplementary Table 10 and Supplementary Figures 16, 17. The findings uncovered a molecular mechanism by which the syntheses of fatty acids, lipids, polyunsaturated fatty acids and eicosanoids, the concentration of colfosceril palmitate and triacylglycerol and the metabolism of fatty acids are increased that is related to the loss of cardiomyocyte-derived BDNF, and the molecular mechanism was involved in the increased body weight seen in MYH6-Cre-BDNF^–/–^ mice.

Furthermore, IPA revealed that 41 inflammation regulatory genes (upregulated: 38 genes; downregulated: 3 genes; Supplementary Tables 11–20) were involved in regulating the increase in inflammatory activity in MYH6-Cre-BDNF^–/–^ hearts compared to WT hearts. The increased biofunctions related to these 41 genes were as follows: inflammatory response (upregulated: 25 genes; downregulated: 1 gene; Supplementary Table 11), recruitment of phagocytes (upregulated: 13 genes; downregulated: 1 gene; Supplementary Table 12), chemotaxis of neutrophils (upregulated: 11 genes; Supplementary Table 13), granulocytes (upregulated: 12 genes; Supplementary Table 14) and leukocytes (upregulated: 16 genes; Supplementary Table 15) as well as activation of leukocytes (upregulated: 19 genes; downregulated: 1 gene; Supplementary Table 16), macrophages (upregulated: 9 genes; downregulated: 1 gene; Supplementary Table 17), mononuclear leukocytes (upregulated: 12 genes; Supplementary Table 18), phagocytes (upregulated: 12 genes; downregulated: 1 gene; Supplementary Table 19) and lymphocytes (upregulated: 11 genes; Supplementary Table 20). The interaction networks of the included genes for individual increased biofunctions are shown in Supplementary Figures 18–27. The findings suggested that loss of cardiomyocyte-derived BDNF increased the inflammatory response and activity in the myocardium by increasing chemotaxis of neutrophils, granulocytes, phagocytes, and leukocytes and by activating leukocytes, macrophages, mononuclear leukocytes, phagocytes and lymphocytes. The molecular mechanisms involved in these processes are controlled by the identified genes and their interaction networks, as shown in Supplementary Table 14 and Supplementary Figures 18–27. These results provide one of the possible mechanisms underlying the cardiomyocyte death and degeneration, interstitial fibrosis, and thrombi in the atrial appendages, atria and ventricles as well as the increased inflammation seen in MYH6-Cre-BDNF^–/–^ hearts.

## Discussion

**FIGURE 8:**
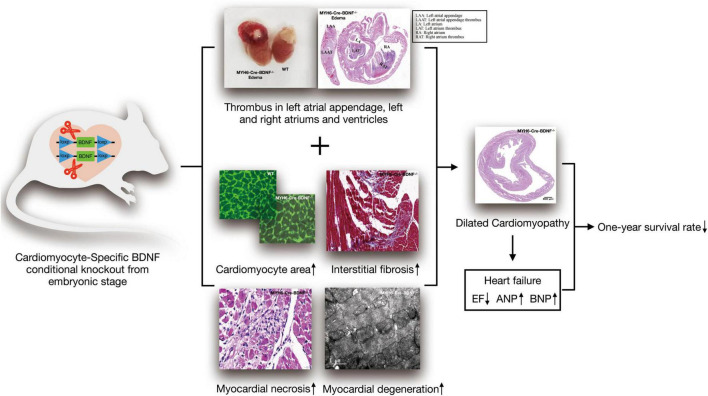
Schematic diagram of the ablation of cardiomyocyte-derived BDNF during development resulting in cardiac degeneration pathology, heart failure and low one-year survival rate.

Currently, the physiopathological role of cardiomyocyte-derived BDNF in the heart is still not precisely known. In the present study, as Figure 8 shown, we established cardiomyocyte-specific BDNF conditional knockout mice (MYH6-Cre-BDNF^–/–^) in which BDNF is ablated specifically in cardiomyocytes upon activation of the myosin heavy chain 6 (MYH6) promoter during development. The MYH6-Cre-BDNF^–/–^ mouse line is able to normally develop, grow and produce offspring. This suggests that ablation of cardiomyocyte-derived BDNF during the development of cardiomyocytes in the embryonic stage can be compensated to maintain whole-body development, growth and reproduction. However, in young adult, 3-month-old MYH6-Cre-BDNF^–/–^ mice, cardiac function was significantly decreased, as we found that the LVEF and LVFS of MYH6-Cre-BDNF^–/–^ hearts declined significantly compared to those of WT hearts. In addition, pathological dilation of the left ventricle was found in 3-month-old MYH6-Cre-BDNF^–/–^ mouse hearts, which was confirmed by their significantly increased LVIDs, LVIDd, LVESV and LVEDV compared to those of WT hearts. Moreover, from 3 months of age onwards, some MYH6-Cre-BDNF^–/–^ mice had somatic edema, and these mice died approximately 1 week after osmotic edema occurred. Echocardiography demonstrated that 3-month-old osmotic edema MYH6-Cre-BDNF^–/–^ hearts have worse cardiac function than non-osmotic edema MYH6-Cre-BDNF^–/–^ and WT hearts, which was supported by their significantly worse LVEF, LVFS, LVIDs, LVIDd, LVESV, and LVEDV. Furthermore, the time window of cardiac dysfunction in female MYH6-Cre-BDNF^–/–^ hearts was later than that in male MYH6-Cre-BDNF^–/–^ hearts. The findings suggest that when endogenous BDNF in cardiomyocytes is ablated during the development of cardiomyocytes, the outside source of BDNF is only able to compensate for the physiological requirements of the heart in the embryonic and young adult stages (approximately 1–2 months old); however, the later lack of compensation causes cardiac structural and functional impairment with increasing age, beginning at approximately 3 months old in males and slightly later in females. Additionally, cardiomyocyte-derived BDNF plays an important role in maintaining the integrity of the heart structurally and functionally, and the tolerance for ablation of cardiomyocyte-derived BDNF is better in females than in males. With increasing age, the lack of compensation of ablated cardiomyocyte-derived BDNF is fatal. Indeed, we found that MYH6-Cre-BDNF^–/–^ mice at 3 months of age and older exhibited a HF phenotype with impaired cardiac function, pathological remodeling and osmotic edema and then died, which led to most MYH6-Cre-BDNF^–/–^ mice dying within 1 year after birth.

Cardiac functional analyses combined with histological and cellular analyses showed that ablation of cardiomyocyte-derived BDNF also causes dilated cardiac cardiomyopathy in young adult hearts. This was confirmed by *in vivo* morphological analysis of the heart by echocardiography, which showed that the IVSTs, IVSTd and LVPWTs of 3-month-old MYH6-Cre-BDNF^–/–^ hearts and osmotic edema MYH6-Cre-BDNF^–/–^ hearts were significantly lower than those of WT hearts. Thus, the results indicated that dilated cardiomyopathy occurred. Indeed, the anatomical and histological analyses of isolated hearts both confirmed that the cross-sectional areas of 3-month-old MYH6-Cre-BDNF^–/–^ and somatic edema MYH6-Cre-BDNF^–/–^ hearts were significantly larger than those of WT hearts. Importantly, both WGA immunofluorescence staining for cardiomyocyte area in tissue and analysis of isolated cardiomyocytes at the cellular level showed that the area of cardiomyocytes in 3-month-old MYH6-Cre-BDNF^–/–^ hearts was significantly larger than that in WT hearts. All facts clearly showed that dilated cardiomyopathy associated with cardiomyocyte hypertrophy was one of the pathological phenotypes caused by deletion of cardiomyocyte-derived BDNF in young adult hearts. Intriguingly, our isolated cardiomyocyte analysis also revealed cardiomyocyte hypertrophy in cardiomyocytes, and an increase in the area of cardiomyocyte nuclei was found in 3-month-old MYH6-Cre-BDNF^–/–^ hearts. The exact mechanism regarding this phenotype is still unknown. One explanation might be that this was a parallel change to adapt to the changes associated with dilated cardiomyopathy and other pathological phenotypes seen in MYH6-Cre-BDNF^–/–^ hearts, which require more gene activation to compensate for the ablation of BDNF in cardiomyocytes. In support of this hypothesis, comparison of whole-transcriptome sequencing results between 3-month-old male MYH6-Cre-BDNF^–/–^ hearts and WT hearts identified the activation of many genes, pathways and biofunctions in the myocardium. In addition, an approximately 6% increase in single-nucleus cardiomyocytes was found in adult MYH6-Cre-BDNF^–/–^ hearts. Most adult mammalian cardiomyocytes have two nuclei, and in the adult stage, only single-nucleus cardiomyocytes still maintain the potential for cell division (30–33). The increase in single-nucleus cardiomyocytes in young adult MYH6-Cre-BDNF^–/–^ hearts might suggest that ablation of BDNF in cardiomyocytes might be able to decrease the production of double-nuclei cardiomyocytes from single-nucleus cardiomyocytes *via* an unknown mechanism that is probably related to maintaining proliferation potential to compensate for pathological remodeling and cardiomyocyte death. The exact cellular and molecular mechanisms need to be further studied in the future.

Cardiomyocyte death, degenerative changes in the myocardium and left atrial appendage thrombus are other notable pathological phenotypes seen in young adult MYH6-Cre-BDNF^–/–^ hearts. Our histological analysis clearly demonstrated focal cardiomyocyte death, focal degenerative changes in cardiomyocytes, focal loss of nuclei in cardiomyocytes, focal inflammation, perivascular fibrosis and focal interstitial fibrosis in young adult MYH6-Cre-BDNF^–/–^ hearts but not in WT hearts, while these pathological phenotypes were found to be more severe in same-age osmotic edema MYH6-Cre-BDNF^–/–^ hearts and older MYH6-Cre-BDNF^–/–^ hearts. Importantly, TEM ultrastructural analysis identified serious damage to cardiomyocyte structure and mitochondrial pathology (disordered distribution, swelling mitochondria, loss of density of mitochondria and serious mitophagy) in young adult MYH6-Cre-BDNF^–/–^ hearts. The above identified pathological changes are related to the cardiomyocyte death, cardiac dysfunction and cardiomyocyte hypertrophy caused by the ablation of cardiomyocyte-derived BDNF. Therefore, this ablation leads to HF and death in adult MYH6-Cre-BDNF^–/–^ mice. In support of this hypothesis, in addition to cardiomyocyte death, degenerative and damage-related changes in the myocardium, cardiomyocyte hypertrophy and osmotic edema, the levels of HF markers (ANP and BNP) were found to be significantly higher in young adult MYH6-Cre-BDNF^–/–^ myocardium, while HF indices (NT-proBNP, BNP and Galectin-3) were also found to be significantly higher in young adult MYH6-Cre-BDNF^–/–^ mouse serum than in WT mouse serum. Thus, ablation of cardiomyocyte-derived BDNF mediated pathological changes in cardiac structure and function, leading to HF, which was a major reason for MYH6-Cre-BDNF^–/–^ mouse death.

Left atrial appendage thrombosis is a unique pathology found in most osmotic edema MYH6-Cre-BDNF^–/–^ hearts. Some osmotic edema MYH6-Cre-BDNF^–/–^ hearts also exhibited right atrial appendage thrombosis, left and right atrial thrombosis and/or left ventricular thrombosis. The thrombi were consistent with mixed thrombus pathological morphology, which includes infiltration with red blood cells, macrophages, lymphocytes and nodular eosinophilic material. In this study, we still cannot explain the exact reason and mechanism underlying this pathological phenotype. A possible explanation might be attributed to hemodynamic changes incurred by dilated cardiomyopathy and the progression of HF combined with the activation of inflammation and ROS as well as metabolic disorders in the myocardium after BDNF ablation, which were identified via transcriptome sequencing combined with IPA. The underlying molecular mechanism needs to be further studied.

The results of a MI study using MYH6-Cre-BDNF^–/–^ mice clearly demonstrated that ablation of cardiomyocyte-derived BDNF in adult hearts was deleterious regardless of cardiac function and regeneration when young adult hearts experience MI. This suggests that endogenous BDNF in cardiomyocytes is indispensable in maintaining the physiological integrity of cardiac structure and function as well as in healing and regeneration of ischemic myocardial pathology. In support of this hypothesis, we found that the serum level of BDNF in MYH6-Cre-BDNF^–/–^ mice was significantly higher than that in WT mice. This result means that BDNF from other cell and tissue sources might fail to compensate for the loss of cardiomyocyte-derived BDNF.

In the present study, the underlying molecular mechanism of cardiac pathology initiated by ablation of cardiomyocyte-derived BDNF was also investigated by whole-transcriptome sequencing of young adult MYH6-Cre-BDNF^–/–^ hearts and parallel WT hearts. IPA is based on an experimentally confirmed reported knowledge database, and the effectors for biofunction, canonical pathways and interaction networks identified by IPA have high reliability; therefore, in the present study, the IPA system was applied to analyze the regulated biofunctions, canonical pathways and interaction networks of genes that were differentially expressed due to BDNF ablation in cardiomyocytes. Indeed, IPA identified pathways and gene interaction networks involved in increased heart apoptosis, activation of cardiac inflammation and ROS, and increased body weight and metabolic disorders in MYH6-Cre-BDNF^–/–^ hearts. Among them, nine genes (upregulated: ADIPOQ, FASH, GAPDH, LCN2, RAC1, and SCD; downregulated: ANGPT1, CXADR, and PPP1R10) and their interaction network were revealed to be related to the increased apoptosis in MYH6-Cre-BDNF^–/–^ hearts. As shown in the interaction network predicted by IPA, these nine genes are able to regulate the activation of heart apoptosis. In addition, IPA revealed that biofunctions involved in the synthesis of reactive oxygen species (ROS) (19 related genes in Table 2) and pathways for the production of nitric oxide and reactive oxygen species in macrophages (upregulated: APOC2, LYZ, NCF1, RAC1, S100A8, and SERPINA1; downregulated: PPP1R10) were activated in MYH6-Cre-BDNF^–/–^ hearts. Furthermore, 41 inflammation regulatory genes were identified to regulate the increase in the inflammatory response in MYH6-Cre-BDNF^–/–^ hearts compared to WT hearts via activation of the following functions (Supplementary Tables 11–20 and Supplementary Figures 18–27): inflammatory response; recruitment of phagocytes; chemotaxis of neutrophils, granulocytes and leukocytes; and activation of leukocytes, macrophages, mononuclear leukocytes, phagocytes and lymphocytes. In addition, the STAT3 pathway (6 genes in Supplementary Figure 15) was activated in MYH6-Cre-BDNF^–/–^ hearts. Hence, increased heart apoptosis, inflammation, ROS production and STAT3 pathway activation regulated by the identified genes and their interaction networks and pathways in MYH6-Cre-BDNF^–/–^ hearts are proposed as possible molecular mechanisms mediating the cardiomyocyte death, cardiac dysfunction and degeneration and HF caused by the ablation of cardiomyocyte-derived BDNF.

Nine genes (Supplementary Table 1) were revealed to be related to an increase in weight gain. Increased lipid metabolism was also found in MYH6-Cre-BDNF^–/–^ hearts compared to WT hearts via activation of the following functions (Supplementary Tables 2–8 and Supplementary Figures 8–14): synthesis of fatty acids, synthesis of lipids, biosynthesis of polyunsaturated fatty acids, synthesis of eicosanoids, concentration of colfosceril palmitate and concentration of triacylglycerol as well as fatty acid metabolism. Furthermore, melatonin degradation I and acetone degradation I (to methylglyoxal) (4 genes in Supplementary Figures 16, 17) were revealed to be activated in MYH6-Cre-BDNF^–/–^ hearts. Hence, increases in weight gain and disordered metabolism of lipids and fatty acids as well as melatonin and acetone degradation regulated by the identified genes and their interaction networks as well as pathways in MYH6-Cre-BDNF^–/–^ hearts are proposed as possible molecular mechanisms to mediate the increases in body weight, inflammation and ROS seen in MYH6-Cre-BDNF^–/–^ mice.

The findings of the present study demonstrate that cardiomyocyte-derived BDNF is irreplaceable for maintaining the integrity of cardiac structure and function in the adult heart and regeneration after MI. It appears that the compensatory effect of non-cardiomyocyte-derived BDNF is merely able to meet the demands of embryonic and young adult (approximately 1–2-month-old) cardiac physiology when endogenous BDNF in cardiomyocytes is ablated. According to the findings from the present study, the pathological phenotypes caused by the lack of compensation for the ablated cardiomyocyte-derived BDNF during the developmental stage will occur in young adult hearts. In a previous report, ablation of cardiomyocyte-derived BDNF using an inducible 2-month-old cardiomyocyte-specific BDNF conditional knockout model did not show effects on cardiac remodeling, cardiac function, or myocardial angiogenesis or infarct size after MI; this can be explained by the increased plasma BDNF levels in non-cardiomyocytes compared to those in WT non-cardiomyocytes. Non-cardiomyocyte-derived BDNF successfully compensates and meets the demand for BDNF to maintain cardiac structure and function during short-term ablation of cardiomyocyte-derived BDNF, in which the heart is still under compensatory conditions (22). However, ablating cardiomyocyte-derived BDNF during development results in a different outcome and is harmful to young adult hearts. In support of this hypothesis, the pathological phenotypes seen in this study and the report using an inducible 2-month-old cardiomyocyte-specific TrkB conditional knockout MI mouse model (blockade of cardiomyocyte and non-cardiomyocyte-derived BDNF signaling), which included decreased cardiac function and myocardial angiogenesis in the infarct border zone and increased infarct size and cardiomyocyte apoptosis (22), clearly suggest that in the adult heart, cardiomyocyte-derived BDNF is irreplaceable for maintaining the integrity of cardiac pathophysiology and regenerating injured myocardium.

In fact, BDNF has been demonstrated to have protective effects against myocardial ischemia through survival-related signaling pathways, including the vascular endothelial growth factor (VEGF) and transient receptor potential canonical (TRPC)3/6 channel pathways (34, 35). Exogenous BDNF was found to promote the migration of Sca-1 cardiac progenitor cells derived from the failing heart and repress cell cycle progression, suggesting its potency to ameliorate HF (36). Exercise training increased BDNF protein in skeletal muscles and the non-infarcted area of the left ventricle after MI, which may contribute to the improvement of muscle dysfunction and cardiac function after MI (12). All these findings are consistent with the results of the present study, which shows that the BDNF-TrkB pathway effectively plays a protective role in adult hearts after MI.

In addition, recent studies have revealed that BDNF plays important roles in cardiac pathology and HF. BDNF attenuates doxorubicin-induced cardiac dysfunction by activating Akt signaling (37), and BDNF protects the heart against septic cardiac dysfunction by reducing oxidative stress and apoptosis via TrkB and activating the eNOS/NO pathway (38). Low serum BDNF levels were found to be associated with future coronary events and mortality in patients with angina pectoris (39) and in patients with chronic HF (40) as well as higher cardiac death or rehospitalization due to worsening HF in discharged HF patients (41) and was positively correlated with HF severity (42). These findings support the deduction of the present study that cardiomyocyte-derived BDNF plays a critical role in protection against HF.

Taking into consideration of contrary reports, which the mice with Myh6-Cre or Myh6-MerCreMer (Tamoxifen inducible Cre recombinase) might display potential cardiotoxicity (43–48), or no Myh6-Cre induced cardiactoxicity found in many other researches (49–54). And it was reported that the Myh6-Cre mice with similar but mixed genetic background displayed no cardiotoxicity (26, 27). In present study, in order to avoid the potential cardiotoxicity from Myh6-Cre, the mating strategy to generate Myh6-Cre-BDNF*^flox^* mice with mixed background was adopted as shown in methodology. Thus, pathological phenotypes and dysfunction of cardiac function found in present study from our established Myh6-Cre^±^-BDNF*^flox/flox^* mice were attributed to the ablation of BDNF but not the potential cardiotoxicity of Myh6-Cre (Supplementary Figures 28–31).

Due to the limitations of time, model and technique, the function studies of IPA predicted pathways (such as for degeneration pathology and cardiac inflammation, etc.), and the rescue analysis of BDNF for neonatal myocardium were not performed in present study. In addition, due to the reason which it was found the pathological phenotypes that occurred in MYH6-Cre-BDNF^–/–^ mice were not sex dependent; however, the time window of cardiac dysfunction in female MYH6-Cre-BDNF^–/–^ hearts was later than that in male MYH6-Cre-BDNF^–/–^ hearts. In the period of this study, we fail to collect the 3-month-old female MYH6-Cre-BDNF^–/–^ edema mice. Therefore, 3-month-old female MYH6-Cre-BDNF^–/–^ edema mice was not observed. All these issues will be set as our future study.

In summary, the present study demonstrated that ablation of cardiomyocyte-derived BDNF during the developmental stage did not impair embryonic survival, growth or reproduction; however, in young adult hearts, it caused cardiomyocyte death, myocardial degeneration, cardiomyocyte hypertrophy, left atrial appendage thrombosis, decreased cardiac function, increased cardiac inflammation and ROS activity, and metabolic disorders, leading to HF in adult hearts and eventually resulting in a decrease in the one-year survival rate. In addition, ablation of cardiomyocyte-derived BDNF during the developmental stage led to the exacerbation of cardiac dysfunction and poor regeneration after MI in adult hearts. Thus, cardiomyocyte-derived BDNF is irreplaceable for maintaining the integrity of cardiac structure and function in the adult heart and regeneration after MI. Therefore, the BDNF-TrkB pathway will be a novel target for myocardial pathophysiology in the adult heart.

## Data availability statement

The datasets presented in this study can be found in online repositories. The names of the repository/repositories and accession number(s) can be found below: https://www.ncbi.nlm.nih.gov/, PRJNA857912.

## Ethics statement

This animal study was reviewed and approved by the Jinan University Animal Care Committee.

## Author contributions

LL, HG, and BL performed most of the experiments and analyzed the data. CL, WG, YC, HC, XZ, and YL contributed to knockout animal breeding and part of the data collection. ZY, RH, ZZ, LYL, HZ, QP, and XQ contributed to the discussion. DC conceived and designed this work and wrote the manuscript. All authors contributed to the article and approved the submitted version.

## Footnotes

1
http://www.ingenuity.com
