# Supplementary material for: Ablation of cardiomyocyte-derived BDNF during development causes myocardial degeneration and heart failure in the adult mouse heart
Source: Front Cardiovasc Med. 2022 Aug 18;9:967463. doi: 10.3389/fcvm.2022.967463 (PMC9433718; doi:10.3389/fcvm.2022.967463)
Supplement: Supplementary file 1 [file Data_Sheet_1.pdf]

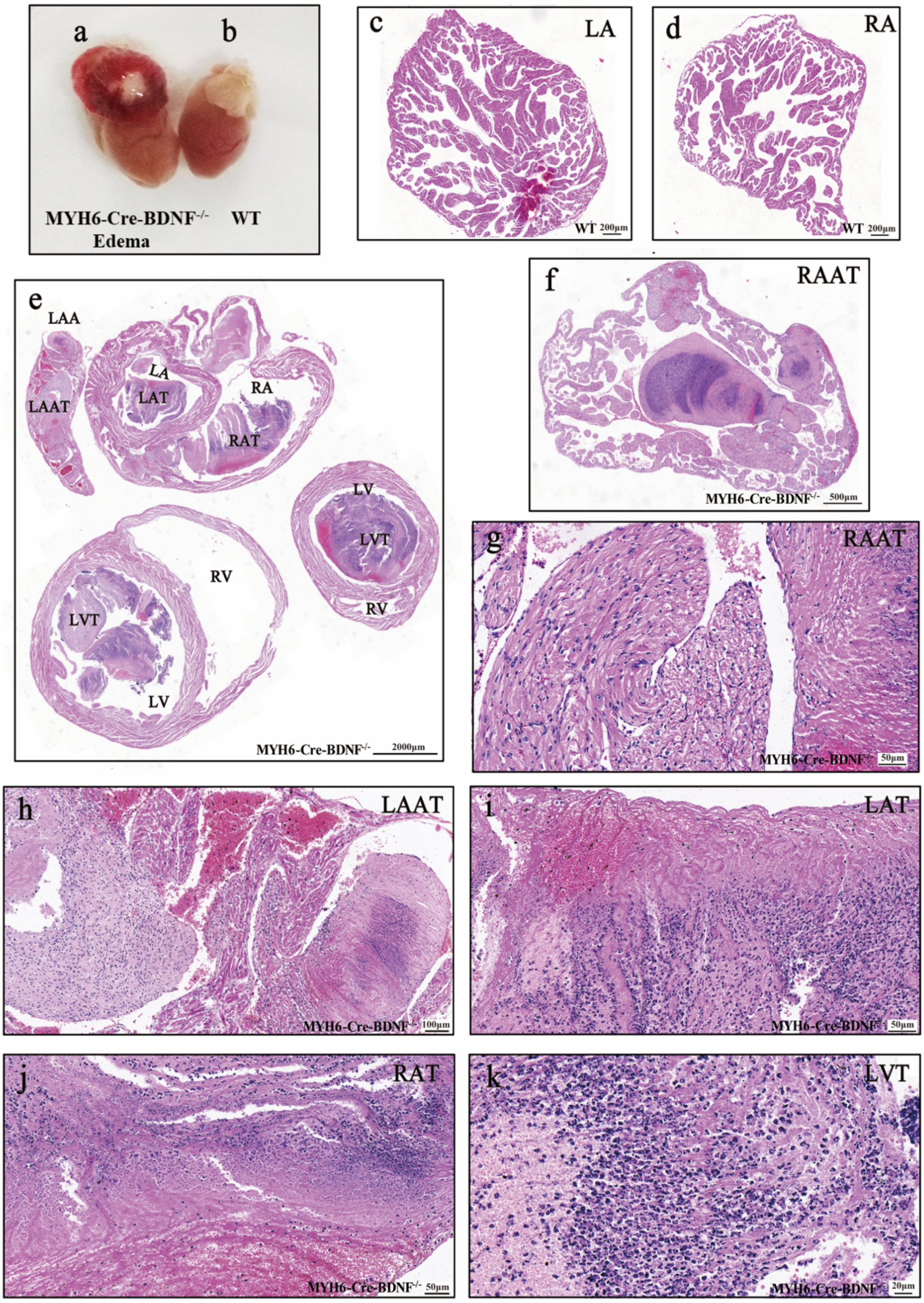

Supplementary Figure 1. Left atrial appendage, right atrial appendage, left atrial, right atrial, and left ventricular thromboses are found in young adult MYH6-Cre-BDNF$^{-/-}$ hearts: a: Representative morphology of left atrial appendage thrombus seen in MYH6-Cre-BDNF$^{-/-}$ hearts. b: Representative normal morphology

of the left atrial appendage seen in WT hearts. c: Representative H&E staining of the left atrial appendage in wild-type hearts. d: Representative H&E staining of the right atrial appendage in wild-type hearts. e: Representative H&E staining of MYH6-Cre-BDNF$^{-/-}$ hearts with left atrial appendage, left atrial, right atrial, and left ventricular thromboses in low-power view. f: Representative H&E staining of MYH6-Cre-BDNF$^{-/-}$ hearts with right atrial appendage thrombus in low-power view. g: Representative H&E staining of MYH6-Cre-BDNF$^{-/-}$ hearts with right atrial appendage thrombus in high-power view. h–j: Representative H&E staining of MYH6-Cre-BDNF$^{-/-}$ hearts with left atrial appendage, left atrial, left ventricular, right atrial appendage and right atrial thromboses in high-power view. H&E histological staining revealed a left atrial appendage thrombus in some 3-month-old MYH6-Cre-BDNF$^{-/-}$ hearts and most osmotic edema MYH6-Cre-BDNF$^{-/-}$ hearts (a, e, h). Some osmotic edema MYH6-Cre-BDNF$^{-/-}$ hearts, in addition to left atrial appendage thrombus, also exhibited right atrial appendage, left and right atrial and left ventricular thromboses (e, f, i–j). In addition, H&E staining revealed that the thrombus had a mixed thrombus pathological morphology that was infiltrated with red blood cells, macrophages, lymphocytes and nodular eosinophilic material (h–k). LAA: Left atrial appendage. RAA: Right atrial appendage. LA: Left atrium. RA: Right atrium. LV: Left ventricle. RV: Right ventricle. LAAT, RAAT, LAT, RAT, and LVT: LAA, RAA, LA, RA and LV thromboses. WT: Wild type. MYH6-Cre-BDNF$^{-/-}$: Cardiomyocyte-derived BDNF conditional knockout. n=3–12. Bar size as shown in the figure.

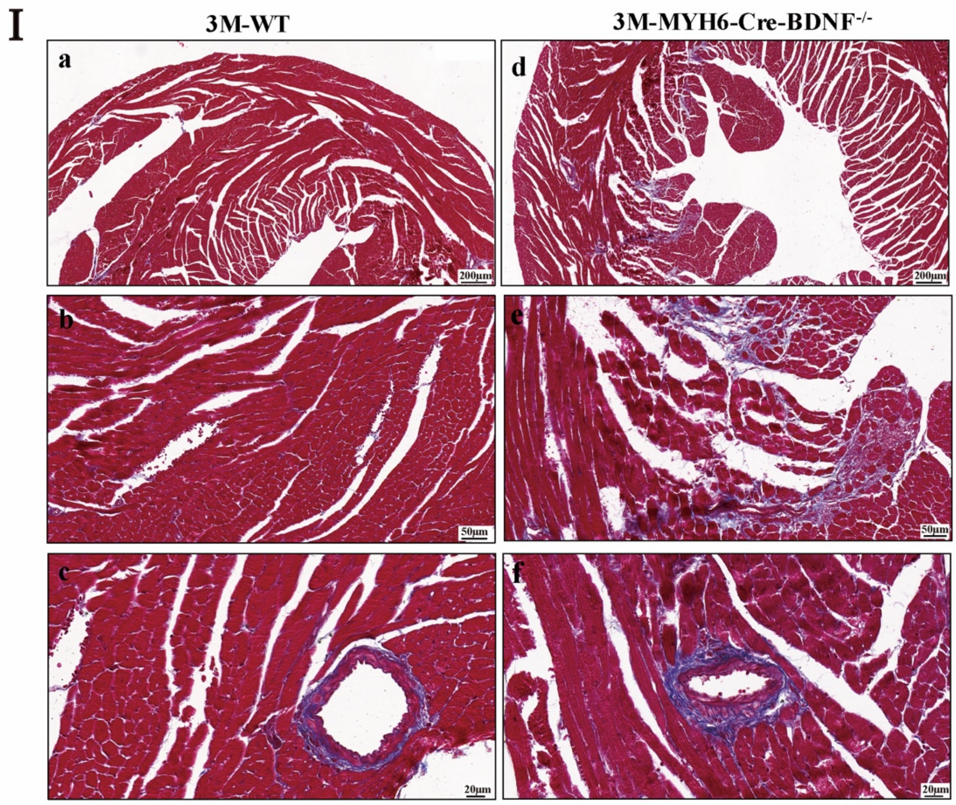

II

3M-WT	3M-MYH6-Cre-BDNF$^{-/-}$
−	+

Supplementary Figure 2. Interstitial fibrosis is found in young adult MYH6-Cre-BDNF$^{-/-}$ hearts: I： Masson’s trichrome staining revealed that compared to WT hearts (a–c), 3-month-old MYH6-Cre-BDNF$^{-/-}$ myocardium showed more extensive perivascular fibrosis (d, e) and focal scatter interstitial fibrosis (d, f) in the myocardium, mainly located in the inner myocardium. **II:** Semi-quatitation of I. n=3. Bar size as shown in the figure.

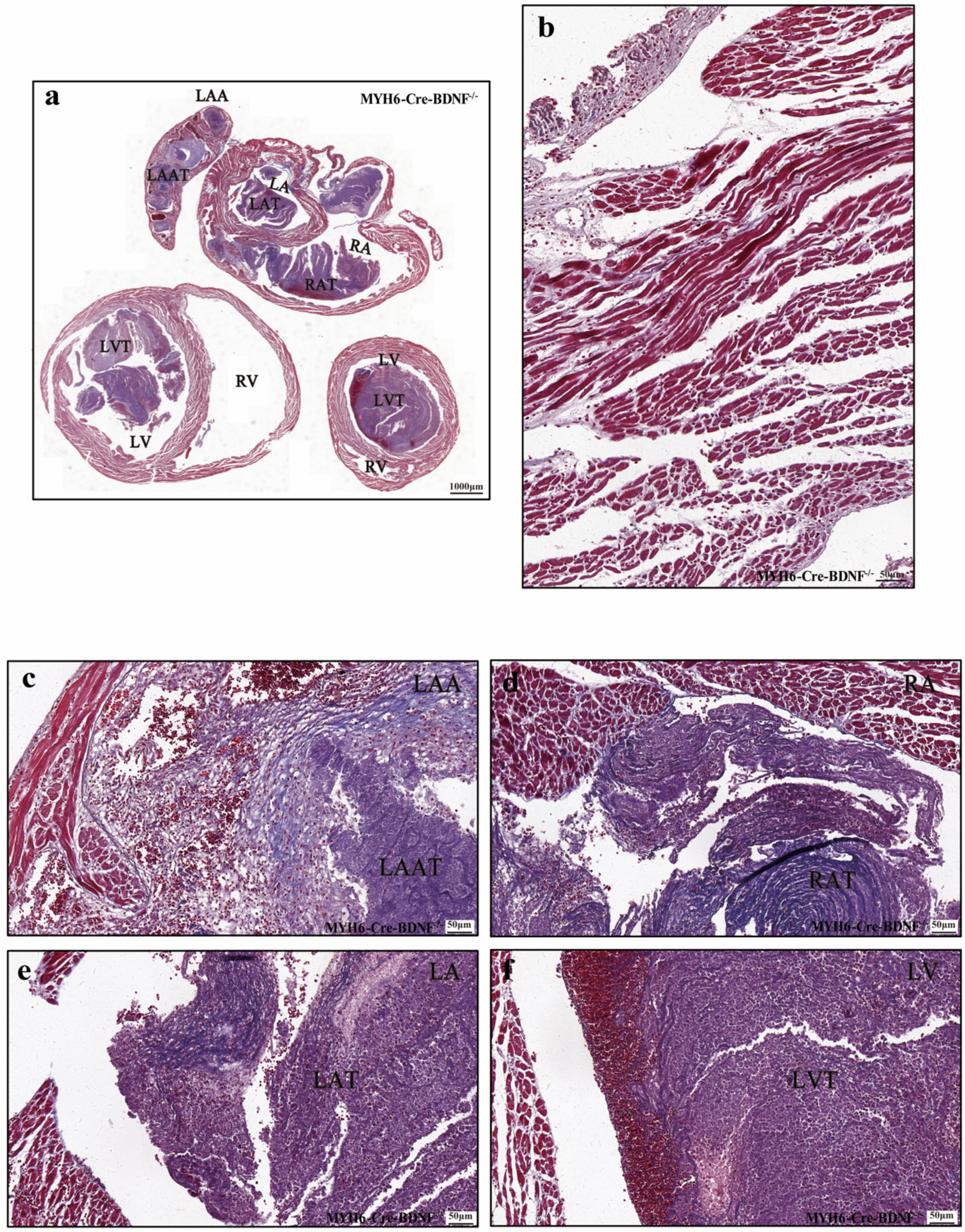

Supplementary Figure 3. Extensive interstitial fibrosis was found in the myocardium and the left atrial appendage, left atrial, right atrial, and left ventricular thromboses formed in 3-month-old osmotic edema MYH6-Cre-BDNF$^{-/-}$ hearts: a: Representative Masson's trichrome staining of 3-month-old osmotic edema MYH6-Cre-BDNF$^{-/-}$ heart in the formed left atrial appendage, left atrial, right atrial, and left ventricular thromboses in low-power view (a), in the left

ventricle (b), in formed left atrial appendage thrombus (c), in formed right atrium thrombus (d), in formed left atrium thrombus (e), and in formed left ventricle thrombus (f). More extensive interstitial fibrosis was found in the myocardium and thrombus, which were located in the atrial appendage and atrial and ventricular cavity, compared to 3-month-old MYH6-Cre-BDNF$^{-/-}$ hearts and WT hearts, as shown in Fig.S2. LAA: Left atrial appendage. LA: Left atrium. RA: Right atrium. LV: Left ventricle. RV: Right ventricle. LAAT, LAT, RAT, and LVT: LAA, RAA, LA, RA and LV thromboses. MYH6-Cre-BDNF$^{-/-}$: Cardiomyocyte-derived BDNF conditional knockout. n=5. Bar size as shown in the figure.

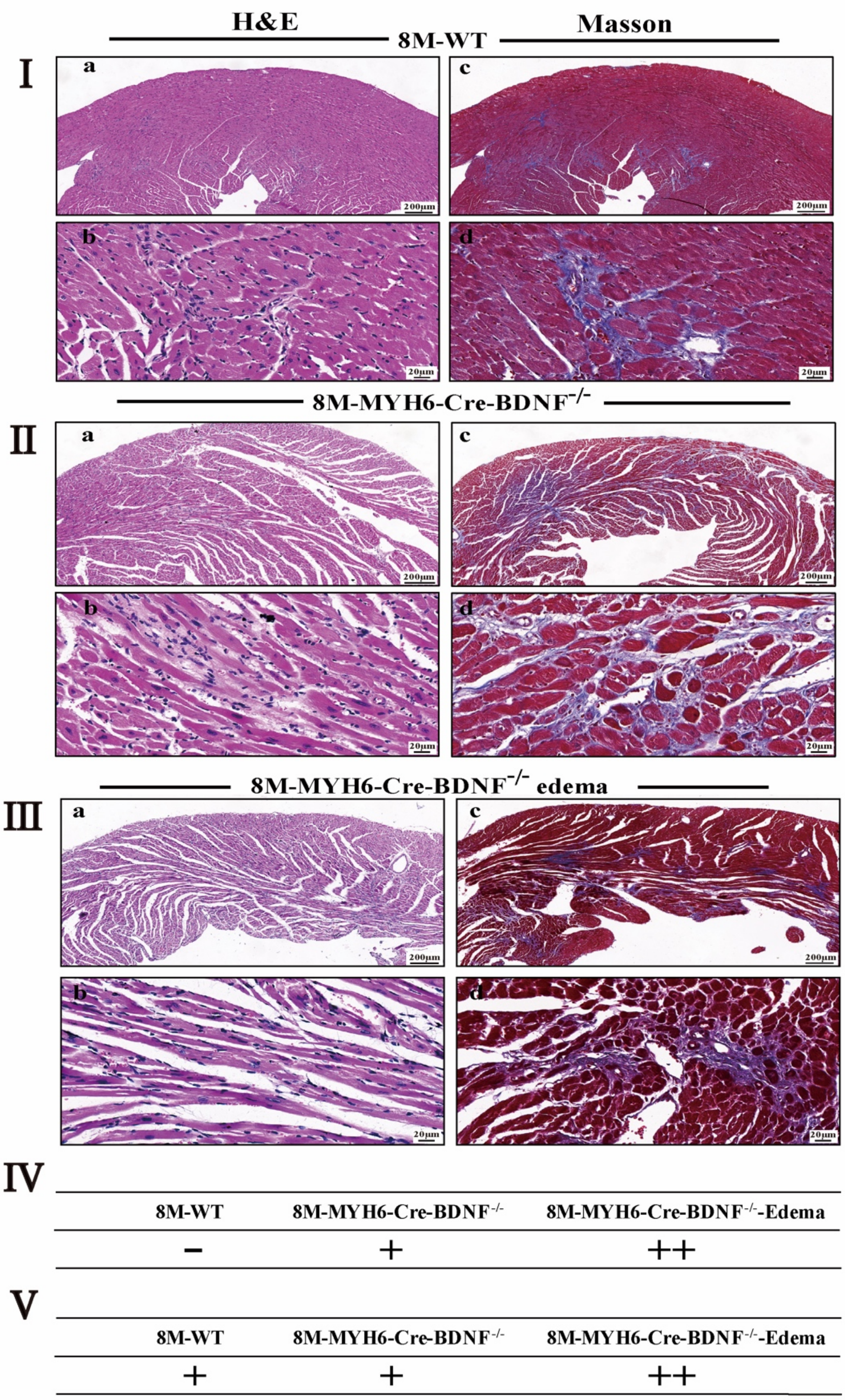

Supplementary Figure 4. More extensive cardiomyocyte death and interstitial fibrosis are found in aged MYH6-Cre-BDNF$^{-/-}$ hearts: Representative H&E staining and Masson's trichrome staining of 8-month-old WT hearts (I), 8-month-old

MYH6-Cre-BDNF$^{-/-}$ hearts (II) and 8-month-old osmotic edema MYH6-Cre-BDNF$^{-/-}$ hearts (III). More extensive cardiomyocyte loss, cardiomyocyte atrophy and interstitial fibrosis were found in 8-month-old MYH6-Cre-BDNF$^{-/-}$ myocardium than in 8-month-old WT myocardium. In addition, more extensive cardiomyocyte loss and cardiomyocyte atrophy were found in 8-month-old osmotic edema MYH6-Cre-BDNF$^{-/-}$ myocardium than in 8-month-old MYH6-Cre-BDNF$^{-/-}$ myocardium and 8-month-old WT myocardium. Furthermore, more extensive cardiomyocyte loss, cardiomyocyte atrophy and interstitial fibrosis were found in 8-month-old MYH6-Cre-BDNF$^{-/-}$ myocardium than in 3-month-old MYH6-Cre-BDNF$^{-/-}$ myocardium, as shown in Fig.4b&c and Fig.S2d–f. **IV:** Semi-quatitation of H&E for Ia,b, IIa,b and IIIa,b. **V:** Semi-quatitation of Masson for Ic,d, IIc,d and IIIc,d. H&E: H&E staining. Masson: Masson's trichrome staining. MYH6-Cre-BDNF$^{-/-}$: Cardiomyocyte-derived BDNF conditional knockout. 8M: 8-month-old. WT: Wild type. n=5. Bar size as shown in the figure.

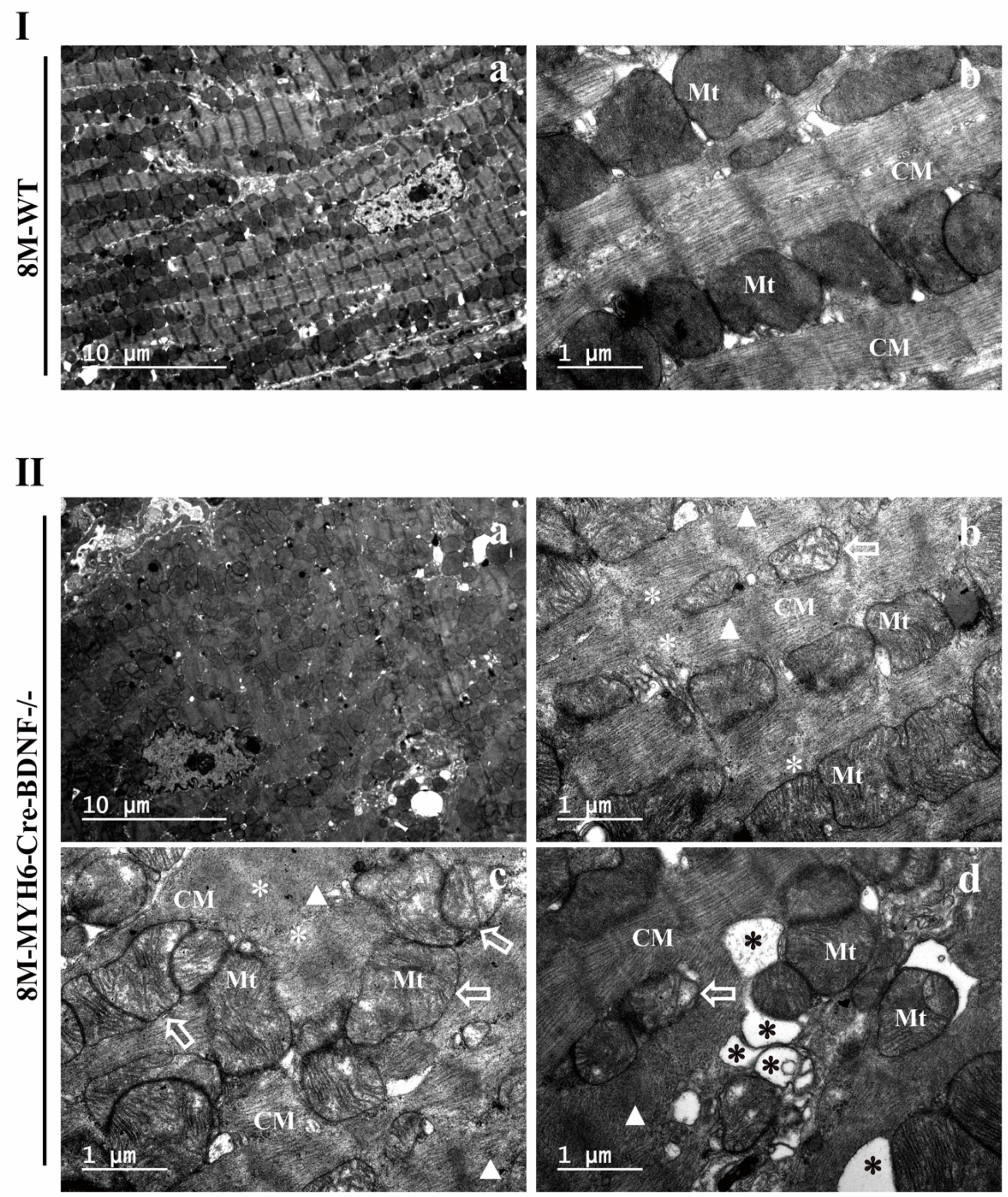

Supplementary Figure 5. Transmission electron microscopy reveals serious pathological changes in mitochondria and myocardium in young adult MYH6-Cre-BDNF$^{-/-}$ hearts: Transmission electron microscopy showed significant mitochondrial distribution disorder, swelling mitochondria, loss of mitochondrial density (II; white open arrow) and serious mitophagy (IId; black asterisk), as well as blurred myofilament structures in cardiomyocytes (IIb,c; white asterisk), unclear sarcomere structures and Z lines, and unclear boundaries between the dark zone and

the bright zone (IIa–c; white triangle) in 8-month-old MYH6-Cre-BDNF$^{-/-}$ hearts. MYH6-Cre-BDNF$^{-/-}$: Cardiomyocyte-derived BDNF conditional knockout. 8M: 8-month-old. WT: Wild type. Mt: Mitochondria. CM: Cardiomyocyte. White triangle: Unclear sarcomere structure and Z line and unclear boundary between the dark zone and the bright zone. White open arrow: Mitochondrial distribution disorder, swelling mitochondria and loss of density of mitochondria. White asterisk: Blurred myofilament structure in cardiomyocytes. Black asterisk: Mitophagy. n=3. Bar size as shown in the figure.

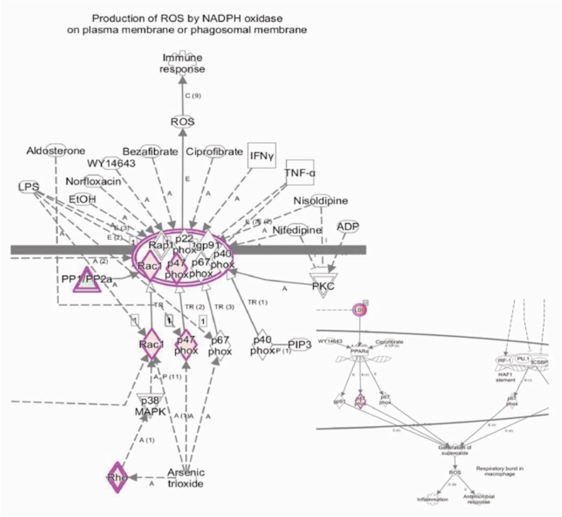

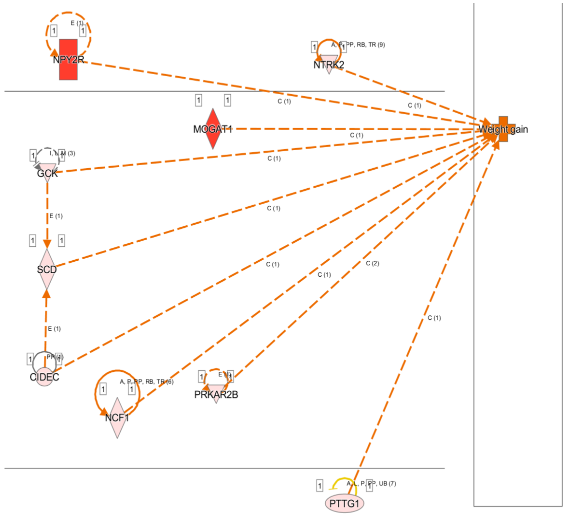

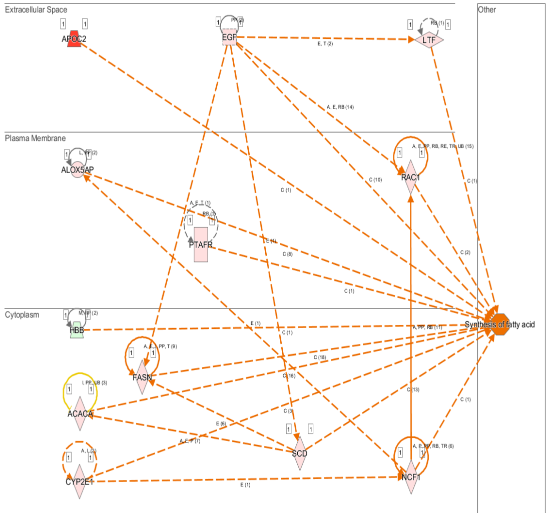

Figure S6 **Figure S7** **Figure S8**

Supplementary Figure 6-8. Activated regulation of production of nitric oxide and reactive oxygen species in macrophage played a role in the heart failure induced by loss of cardiomyocyte derived BDNF (S6): The integrated IPA analysis revealed that the production of nitric oxide and reactive oxygen species in macrophage in cardiomyocyte BDNF conditional knockout heart was enhanced compared with wild type heart (p-value = 3.51E-02; activation z-score = 2.646). The interaction network demonstrates that 7 up-regulated genes were included as shown in the interaction network for the activation of production of nitric oxide and reactive oxygen species in macrophage in cardiomyocyte BDNF conditional knockout heart. **Activated regulation of weight gain in cardiomyocyte BDNF conditional knockout mice (S7):** The integrated

IPA analysis predicted that the weight gain in cardiomyocyte BDNF conditional knockout mice was enhanced compared with wild type mice (p-value = 7.67E-05; activation z-score = 2.530). The interaction network demonstrates that 9 up-regulated genes were included as shown in the interaction network for the activation of weight gain in cardiomyocyte BDNF conditional knockout mice. **Activated regulation of synthesis of fatty acid in cardiomyocyte BDNF conditional knockout heart (S8):** The integrated IPA analysis revealed that the synthesis of fatty acid in cardiomyocyte BDNF conditional knockout heart was enhanced compared with wild type heart (p-value = 4.47E-07; activation z-score = 2.231). The interaction network demonstrates that 12 genes (11 up-regulated genes and 1 down-regulated gene) were included as shown in the interaction network for the activation of synthesis of fatty acid in cardiomyocyte BDNF conditional knockout heart.

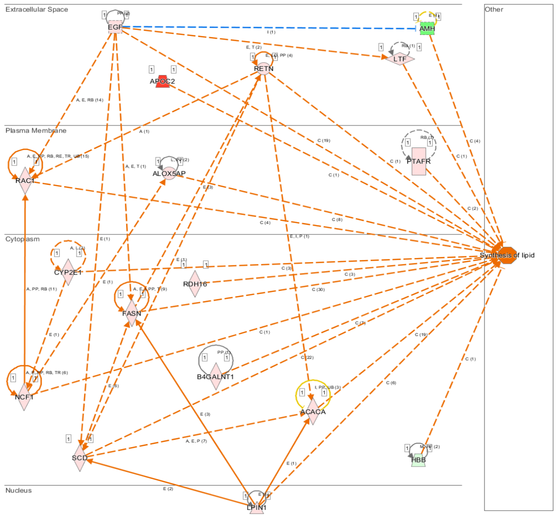

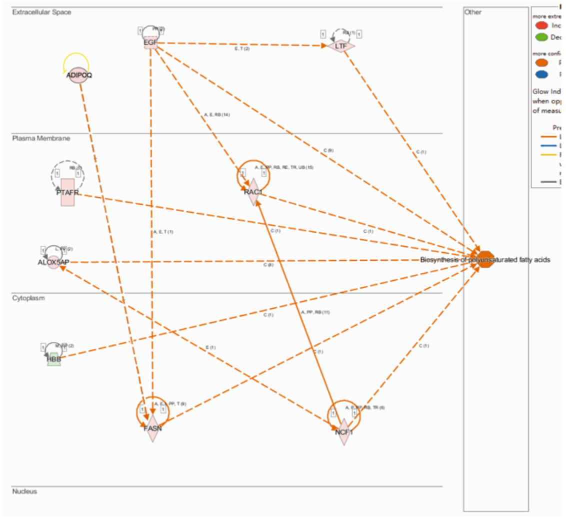

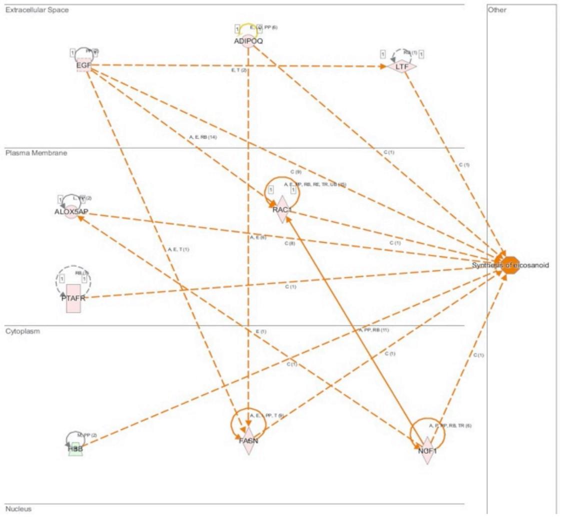

Figure S9 **Figure S10** **Figure S11**

Supplementary Figure 9-11. Activated regulation of synthesis of lipid in cardiomyocyte BDNF conditional knockout heart (S9): The integrated IPA analysis revealed that the synthesis of lipid in cardiomyocyte BDNF conditional knockout heart was enhanced compared with wild type heart (p-value = 4.63E-06; activation z-score = 2.317). The interaction network demonstrates that 17 molecules (15 up-regulated genes and 2 down-regulated genes) were included as shown in the interaction network for the activation of synthesis of lipid in cardiomyocyte BDNF conditional knockout heart. **Activated regulation of biosynthesis of polyunsalturated fatty acids in cardiomyocyte BDNF conditional knockout heart (S10):** The integrated IPA analysis predicted that the biosynthesis of polyunsalturated fatty acids in cardiomyocyte BDNF

conditional knockout heart was enhanced compared with wild type heart (p-value = 4.47E-07; activation z-score = 2.231). The interaction network demonstrates that 9 genes (8 up-regulated genes and 1 down-regulated gene) were included as shown in the interaction network for the activation of biosynthesis of polyunsalturated fatty acids in cardiomyocyte BDNF conditional knockout heart. **Activated regulation of synthesis of eicosanoid in cardiomyocyte BDNF conditional knockout heart (S11):** The integrated IPA analysis identified that the synthesis of eicosanoid in cardiomyocyte BDNF conditional knockout heart was enhanced compared with wild type heart (p-value = 4.47E-07; activation z-score = 2.231). The interaction network demonstrates that 9 genes (8 up-regulated genes and 1 down-regulated gene) were included as shown in the regulatory network for the activation of synthesis of eicosanoid in cardiomyocyte BDNF conditional knockout heart.

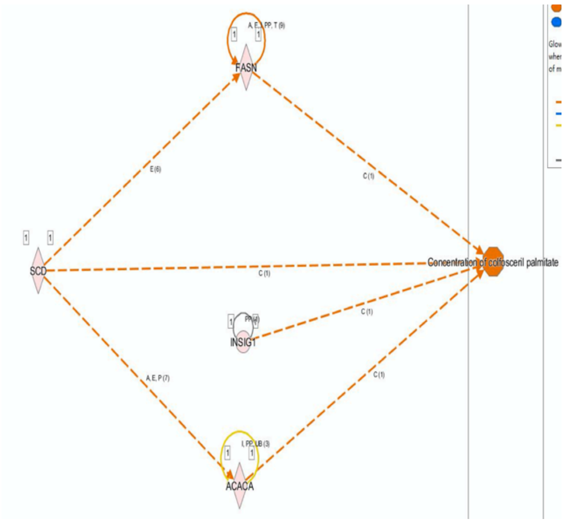

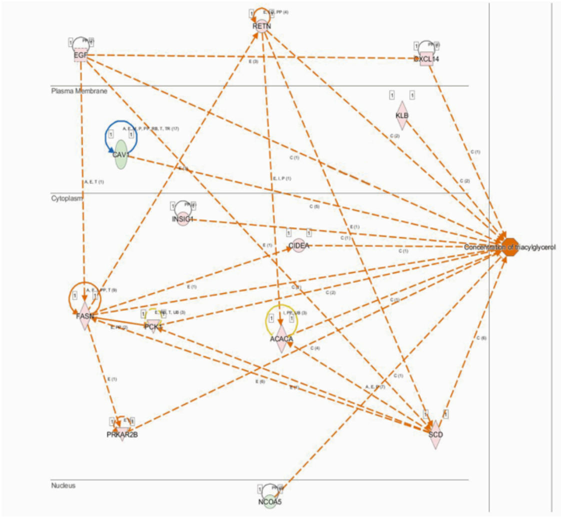

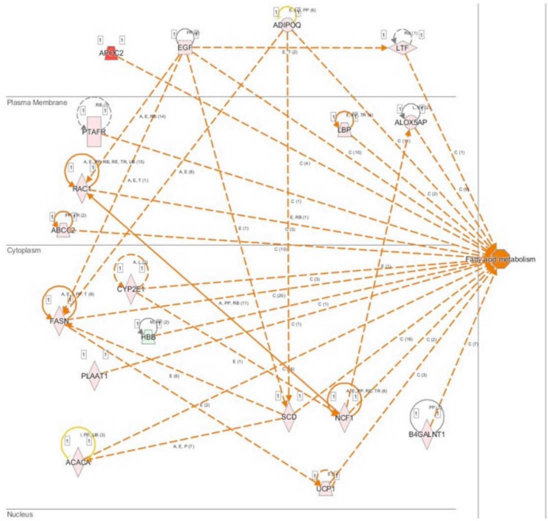

Figure S12 **Figure S13** **Figure S14**

Supplementary Figure 12-14. Activated regulation of concentration of colfosceril palmitate in cardiomyocyte BDNF conditional knockout heart (S12): The integrated IPA analysis revealed that the concentration of colfosceril palmitate in cardiomyocyte BDNF conditional knockout heart was enhanced compared with wild type heart (p-value = 2.35E-06; activation z-score = 2.000). The interaction network demonstrates that 4 up-regulated genes were included as shown in the interaction network for the activation of concentration of colfosceril palmitate in cardiomyocyte BDNF conditional knockout heart. **Activated regulation of concentration of triacylglycerol in cardiomyocyte BDNF conditional knockout heart (S13):** The integrated IPA analysis revealed that the concentration of triacylglycerol in cardiomyocyte

BDNF conditional knockout heart was enhanced compared with wild type heart (p-value = 5.74E-06; activation z-score = 2.246). The interaction network demonstrates that 13 genes (11 up-regulated genes and 2 down-regulated genes) were included as shown in the interaction network for the activation of concentration of triacylglycerol in cardiomyocyte BDNF conditional knockout heart. **Activated of fatty acid metabolism in cardiomyocyte BDNF conditional knockout heart (S14):** The integrated IPA analysis revealed that the fatty acid metabolism in cardiomyocyte BDNF conditional knockout heart was enhanced compared with wild type heart (p-value = 2.46E-10; activation z-score = 2.257). The interaction network demonstrates that 18 genes (17 up-regulated genes and 1 down-regulated gene) were included as shown in the interaction network for the activation of fatty acid metabolism in cardiomyocyte BDNF conditional knockout heart.

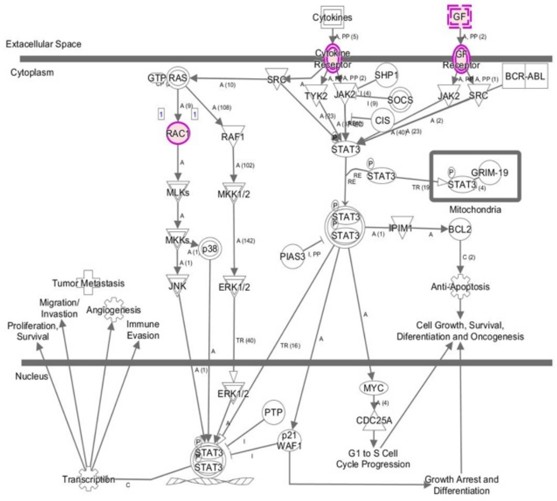

Figure S15

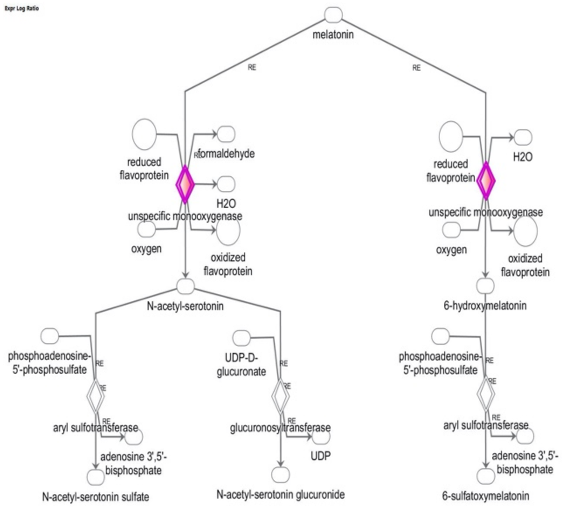

Figure S16

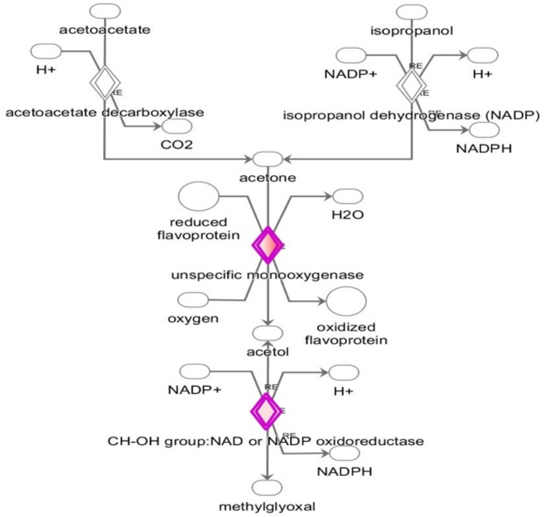

Figure S17

Supplementary Figure 15-17. Activated regulation of STAT3 pathway in cardiomyocyte BDNF conditional knockout heart (S15): The integrated IPA analysis identified that the STAT3 pathway in cardiomyocyte BDNF conditional knockout heart was enhanced compared with wild type heart (p-value = 2.36E-02; activation z-score = 2.000). The interaction network demonstrates that 6 up-regulated genes were included as shown in the interaction network for the activation of STAT3 pathway in cardiomyocyte BDNF conditional knockout heart. **Activated regulation of melatonin degradation I pathway in cardiomyocyte BDNF conditional knockout heart (S16):** The integrated IPA analysis revealed that melatonin degradation I pathway in cardiomyocyte BDNF conditional knockout heart was enhanced compared with wild type heart

(p-value = 1.66E-02; activation z-score = 2.000). The interaction network demonstrates that 4 up-regulated genes were included as shown in the interaction network for the activation of melatonin degradation I in cardiomyocyte BDNF conditional knockout heart. **Activated regulation of acetone degradation I pathway in cardiomyocyte BDNF conditional knockout heart (S17):** The integrated IPA analysis revealed that acetone degradation I pathway in cardiomyocyte BDNF conditional knockout heart was enhanced compared (p=1.37E-03; activation z-score=2) with wild type heart. The interaction network demonstrates that 4 up-regulated genes were included as shown in the interaction network for the activation of acetone degradation I pathway in cardiomyocyte BDNF conditional knockout heart.

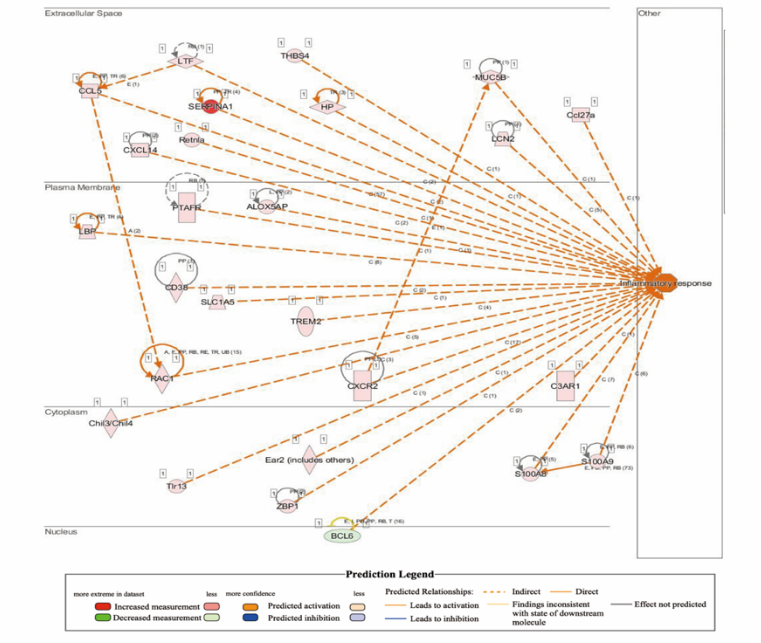

Supplementary Figure 18. Activated regulation of the inflammatory response played a role in heart failure induced by the loss of cardiomyocyte-derived BDNF: Integrated IPA identified that the inflammatory response in cardiomyocyte-derived BDNF conditional knockout hearts was enhanced (p-value = 8.44E-10; activation z-score = 3.513) compared with that in wild-type hearts. The interaction network demonstrated that 26 genes (25 upregulated genes and 1 downregulated gene) were included, as shown in the interaction network for the activation of the inflammatory response in cardiomyocyte-derived BDNF conditional knockout hearts.

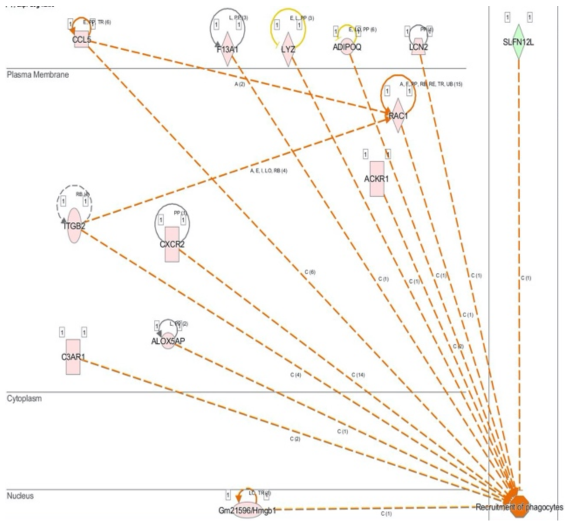

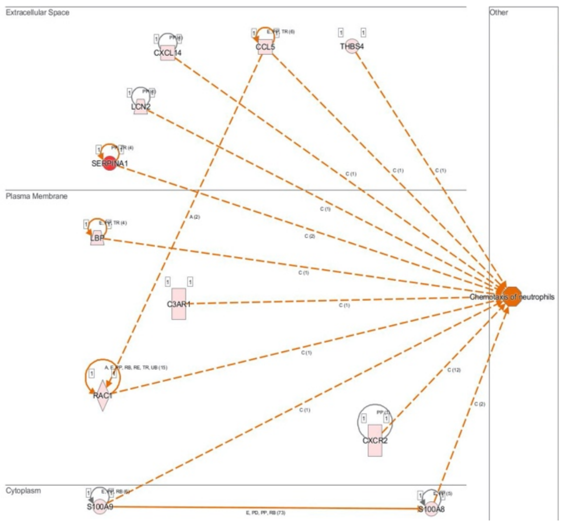

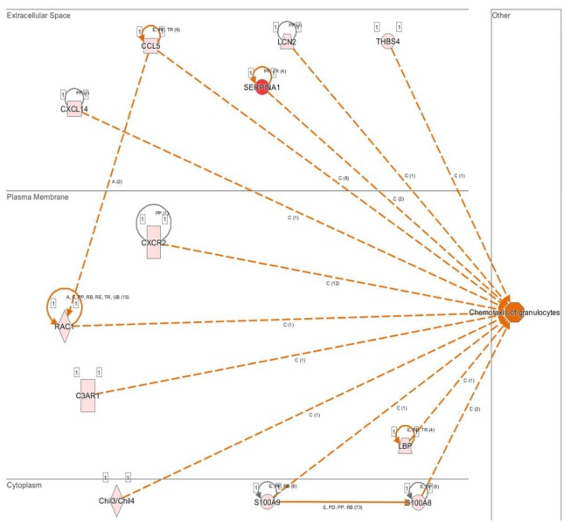

Figure S19 **Figure S20** **Figure S21**

Supplementary Figure 19-21. Activated regulation of recruitment of phagocytes played a role in the heart failure induced by loss of cardiomyocyte derived BDNF (S19): The integrated IPA analysis revealed that the recruitment of phagocytes in cardiomyocyte BDNF conditional knockout heart was enhanced compared with wild type heart (p-value = 2.00E-07; activation z-score = 2.147). The interaction network demonstrates that 13 genes (12 up-regulated genes and 1 down-regulated gene) were included as shown in the interaction network for the activation of recruitment of phagocytes in cardiomyocyte BDNF conditional knockout heart. **Activated regulation of chemotaxis of neutrophils played a role in the heart failure induced by loss of cardiomyocyte derived BDNF (S20):** The integrated IPA analysis identified

that the chemotaxis of neutrophils in cardiomyocyte BDNF conditional knockout heart was enhanced compared with wild type heart (p-value = 1.26E-06; activation z-score = 2.817). The interaction network demonstrates that 11 up-regulated genes were included as shown in the interaction network for the activation of chemotaxis of neutrophils in cardiomyocyte BDNF conditional knockout heart. **Activated regulation of chemotaxis of granulocytes played a role in the heart failure induced by loss of cardiomyocyte derived BDNF (S21):** The integrated IPA analysis revealed that the chemotaxis of granulocytes in cardiomyocyte BDNF conditional knockout heart was enhanced compared with wild type heart (p-value = 2.19E-06; activation z-score = 2.987). The interaction network demonstrates that 12 up-regulated genes were included as shown in the interaction network for the activation of chemotaxis of granulocytes in cardiomyocyte BDNF conditional knockout heart.

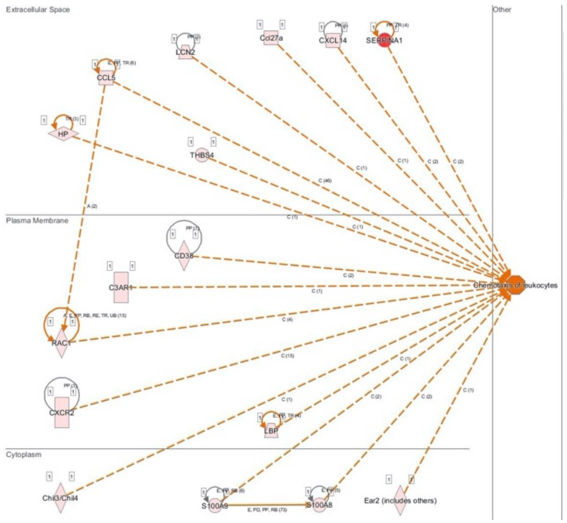

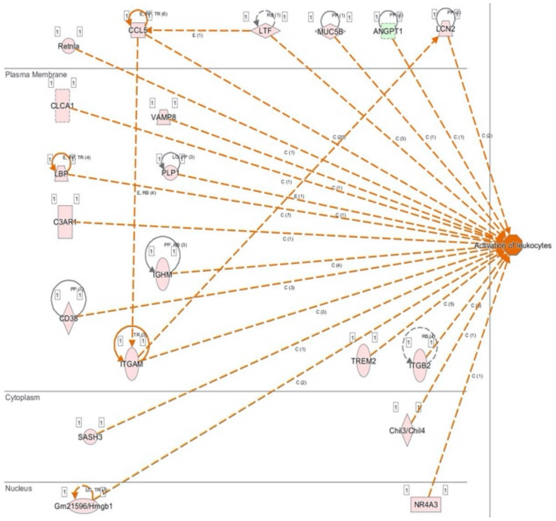

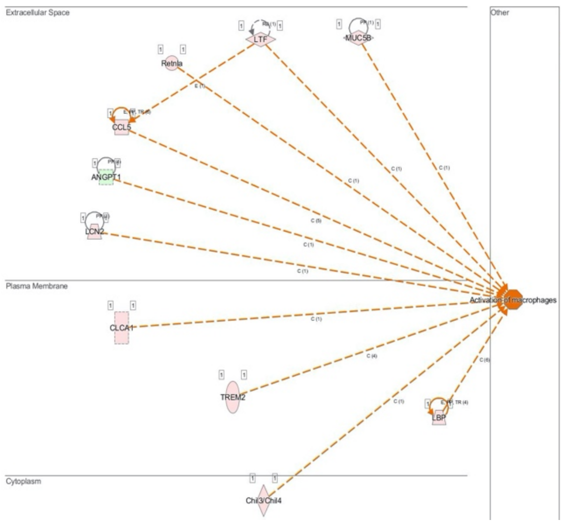

Figure S22 **Figure S23** **Figure S24**

Supplementary Figure 22-24. Activated regulation of chemotaxis of leukocytes played a role in the heart failure induced by loss of cardiomyocyte derived BDNF (S22): The integrated IPA analysis revealed that the chemotaxis of leukocytes in cardiomyocyte BDNF conditional knockout heart was enhanced compared with wild type heart (p-value = 9.78E-06; activation z-score = 2.960). The interaction network demonstrates that 16 up-regulated genes were included as shown in the regulatory network for the activation of chemotaxis of leukocytes in cardiomyocyte BDNF conditional knockout heart. **Activated regulation of leukocytes played a role in the heart failure induced by loss of cardiomyocyte derived BDNF (S23):** The integrated IPA analysis revealed that the activation of leukocytes in cardiomyocyte BDNF

conditional knockout heart was enhanced compared with wild type heart (p-value = 6.09E-05; activation z-score = 3.449). The interaction network demonstrates that 20 genes (19 up-regulated genes and 1 down-regulated gene) were included as shown in the interaction network for the activation of leukocytes in cardiomyocyte BDNF conditional knockout heart. **Activated regulation of macrophages played a role in the heart failure induced by loss of cardiomyocyte derived BDNF (S24):** The integrated IPA analysis identified that the activation of macrophages in cardiomyocyte BDNF conditional knockout heart was enhanced compared with wild type heart (p-value = 2.28E-04; activation z-score = 2.398). The interaction network demonstrates that 10 genes (9 up-regulated genes and 1 down-regulated gene) were included as shwon in the interaction network for the activation of macrophages in cardiomyocyte BDNF conditional knockout heart.

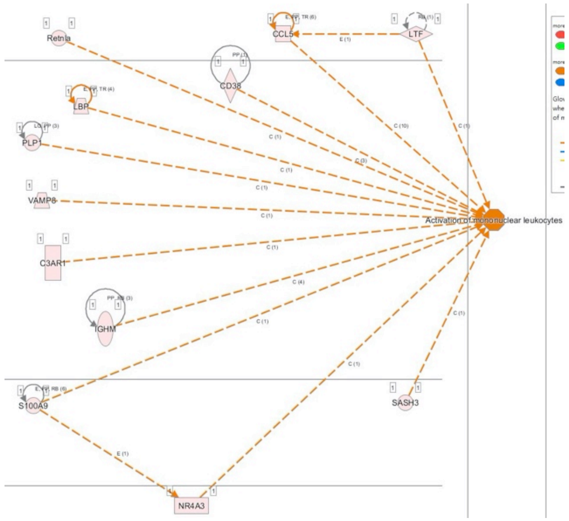

Figure S25

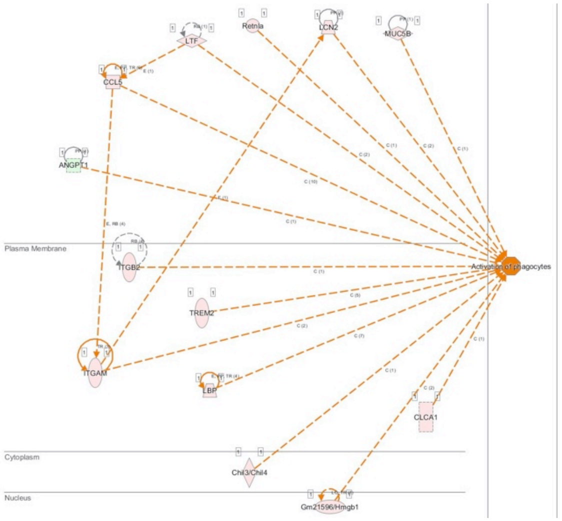

Figure S26

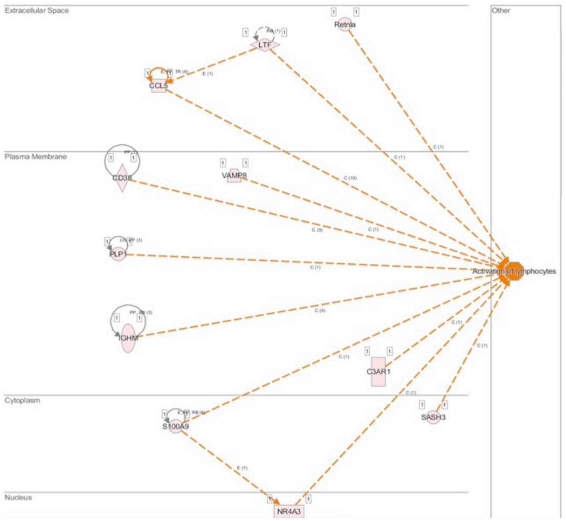

Figure S27

Supplementary Figure 25-27. Activated regulation of mononuclear leukocytes played a role in the heart failure induced by loss of cardiomyocyte derived BDNF (S25): The integrated IPA analysis revealed that the activation of mononuclear leukocytes in cardiomyocyte BDNF conditional knockout heart was enhanced compared with wild type heart (p-value = 3.37E-04; activation z-score = 2.679). The interaction network demonstrates that 12 up-regulated genes were included as shown in the interaction network for the activation of mononuclear leukocytes in cardiomyocyte BDNF conditional knockout heart. **Activated regulation of phagocytes played a role in the heart failure induced by loss of cardiomyocyte derived BDNF (S26):** The integrated IPA analysis revealed that the activation of phagocytes in

cardiomyocyte BDNF conditional knockout heart was enhanced compared with wild type heart (p-value = 3.49E-04; activation z-score = 2.610). The interaction network demonstrates that 13 genes (12 up-regulated genes and 1 down-regulated gene) were included as shown in the interaction network for the activation of phagocytes in cardiomyocyte BDNF conditional knockout heart. **Activated regulation of lymphocytes played a role in the heart failure induced by loss of cardiomyocyte derived BDNF (S27):** The integrated IPA analysis revealed that the activation of lymphocytes in cardiomyocyte BDNF conditional knockout heart was enhanced compared with wild type heart (p-value = 4.02E-04; activation z-score = 2.243). The interaction network demonstrates that 11 up-regulated genes were included as shown in the interaction network for the activation of lymphocytes in cardiomyocyte BDNF conditional knockout heart.

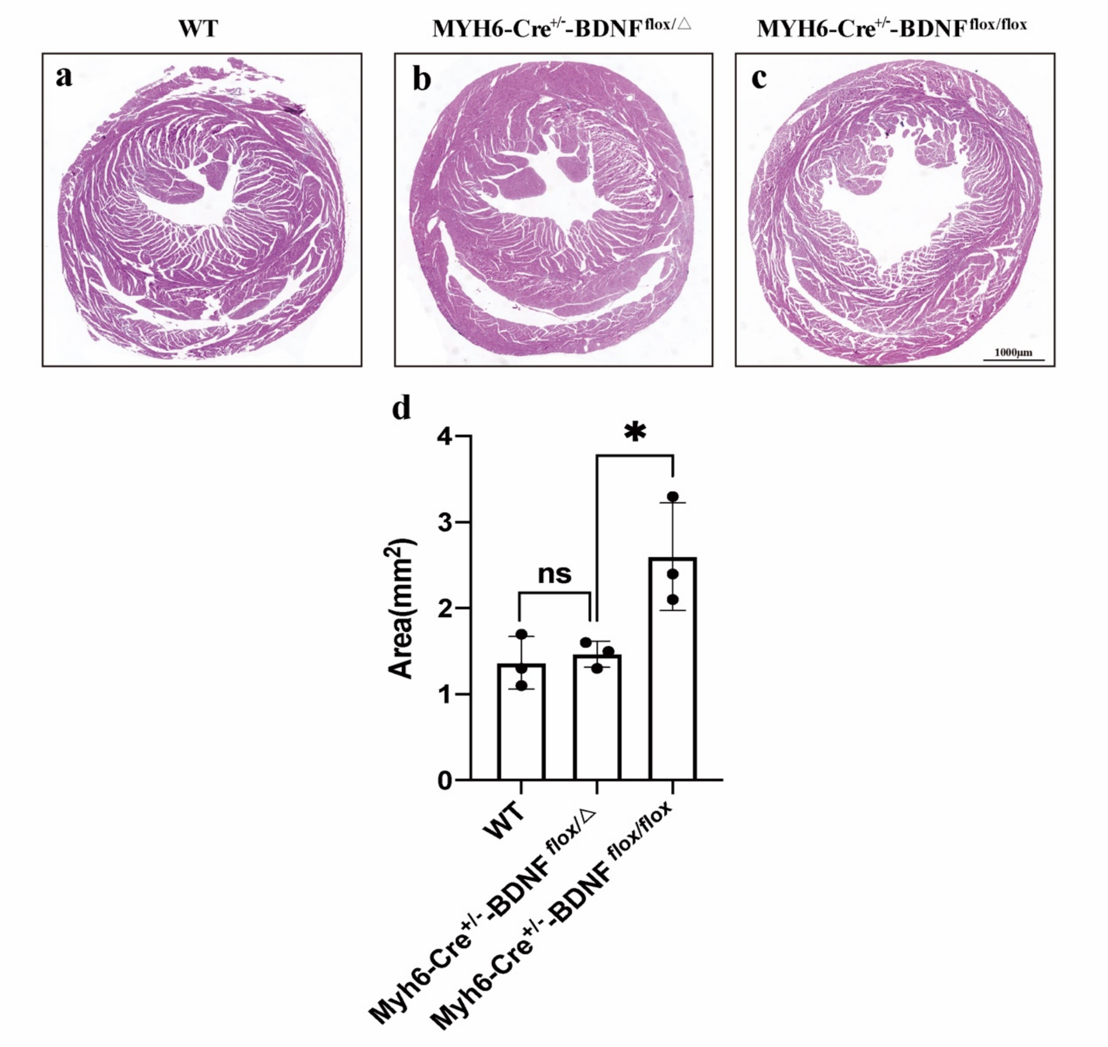

Supplementary Figure 28. The area of left ventricle of 3-month-old MYH6-Cre$^{+/-}$-BDNF$^{flox/\triangle}$ was similar to 3-month-old WT heart. However, the area of left ventricle of 3-month-old MYH6-Cre$^{+/-}$-BDNF$^{flox/flox}$ heart was significantly larger than that of 3-month-old MYH6-Cre$^{+/-}$-BDNF$^{flox/\triangle}$ and WT heart. n=3. *: $p<0.05$.

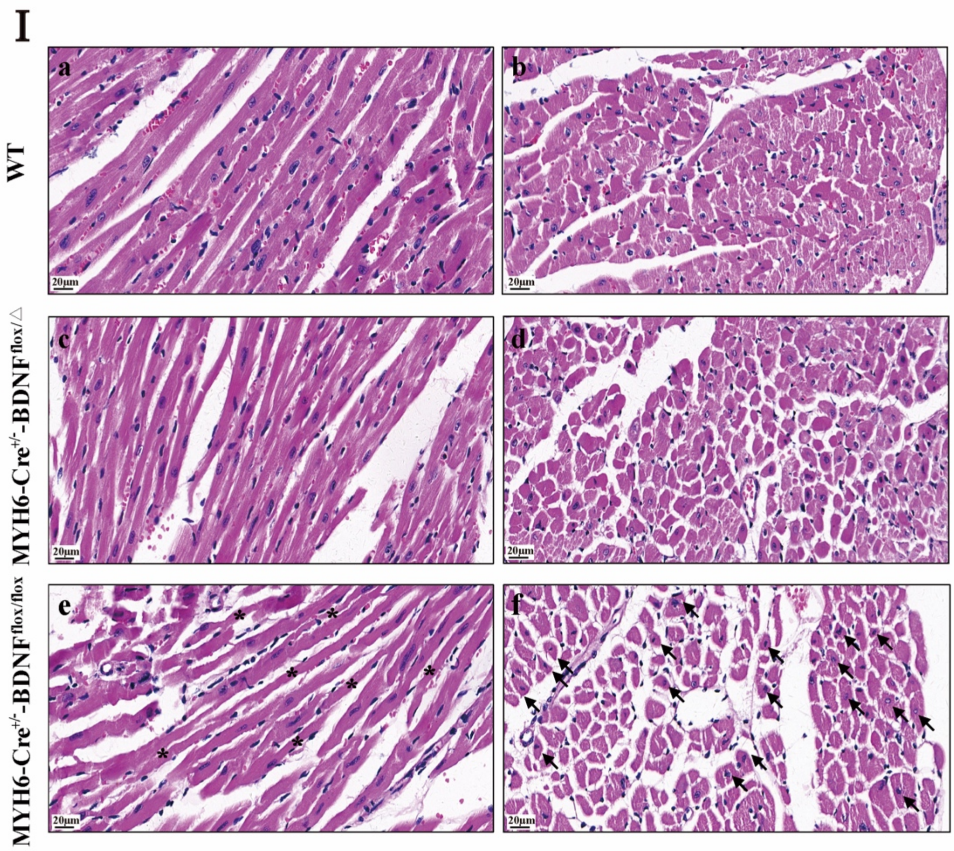

II

WT	MYH6-Cre$^{+/-}$-BDNF$^{flox/\triangle}$	MYH6-Cre$^{+/-}$-BDNF$^{flox/flox}$
−	−	+

Supplementary Figure 29. The morphology of 3-month-old MYH6-Cre$^{+/-}$-BDNF$^{flox/\triangle}$ myocardium was quite similar to 3-month-old WT myocardium. However, degeneration changes in 3-month-old MYH6-Cre$^{+/-}$-BDNF$^{flox/flox}$ myocardium is more serious than those of 3-month-old MYH6-Cre$^{+/-}$-BDNF$^{flox/\triangle}$ myocardium. I: H&E staining revealed that focal cardiomyocyte death (losing cardiomyocytes in e vs. a and c), dilation of cardiomyocyte (asterisk in e vs. a and c) and central nucleus (arrow in f vs. a and c) in 3-month-old MYH6-Cre$^{+/-}$-BDNF$^{flox/flox}$ hearts are more serious than those of 3-month-old MYH6-Cre$^{+/-}$-BDNF$^{flox/flox}$ hearts.

However, the morphology of 3-month-old MYH6-$Cre^{+/-}$-$BDNF^{flox/\triangle}$ myocardium was quite similar to 3-month-old WT myocardium. **II:** Semi-quatitation of I. n=3.

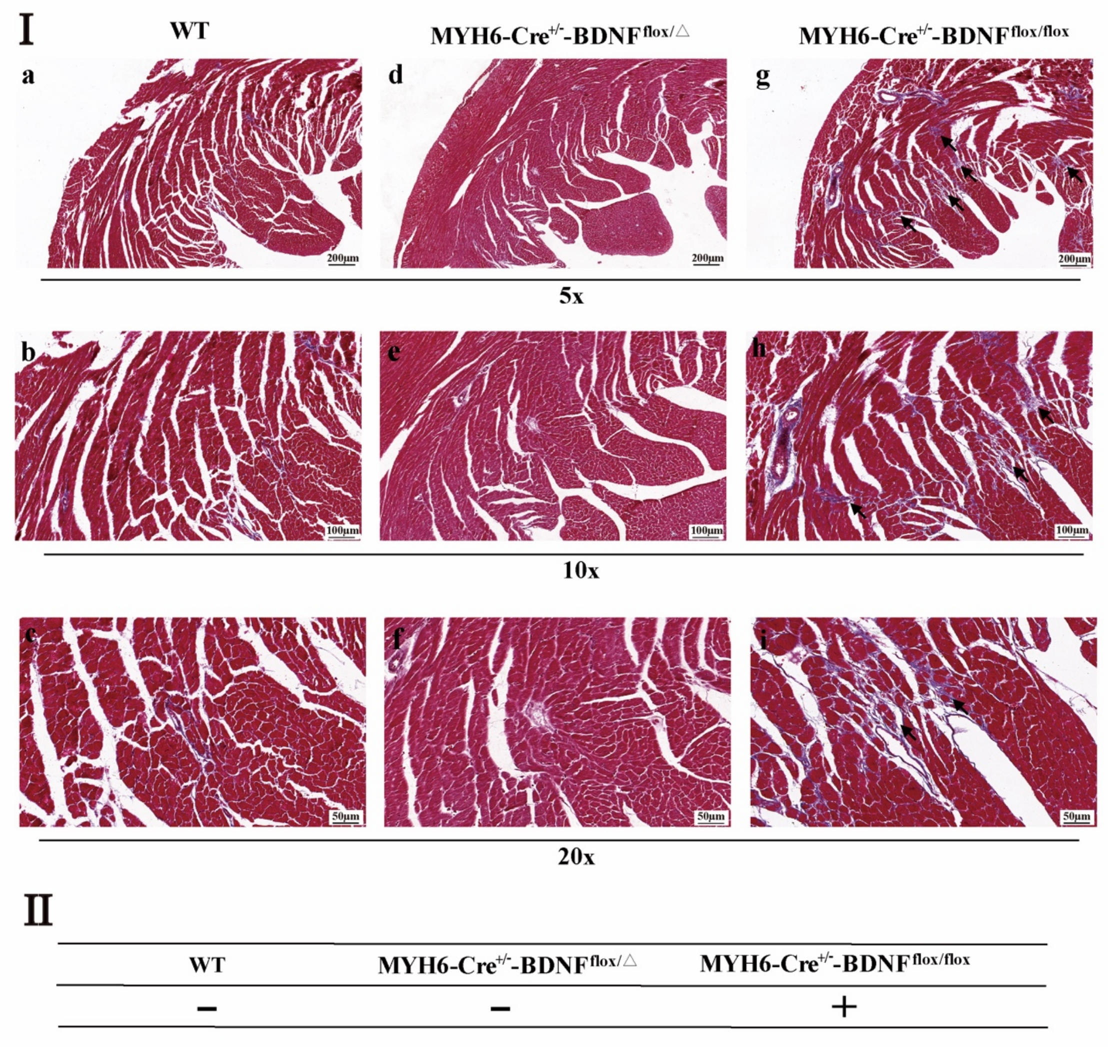

Supplementary Figure 30. There was no significant difference of cardiac fibrosis between 3-month-old MYH6-Cre$^{+/-}$-BDNF$^{flox/\triangle}$ myocardium and 3-month-old WT myocardium. However, cardiac fibrosis in 3-month-old MYH6-Cre$^{+/-}$-BDNF$^{flox/flox}$ myocardium is more serious than those of 3-month-old MYH6-Cre$^{+/-}$-BDNF$^{flox/\triangle}$ myocardium and 3-month-old WT myocardium. I: Massion Trichrome staining revealed that cardiac fibrosis in 3-month-old MYH6-Cre$^{+/-}$-BDNF$^{flox/flox}$ myocardium (g-i) is more serious than those of 3-month-old MYH6-Cre$^{+/-}$-BDNF$^{flox/\triangle}$ myocardium (d-f) and 3-month-old WT myocardium (a-c). **II:** Semi-quatitation of I. n=3.

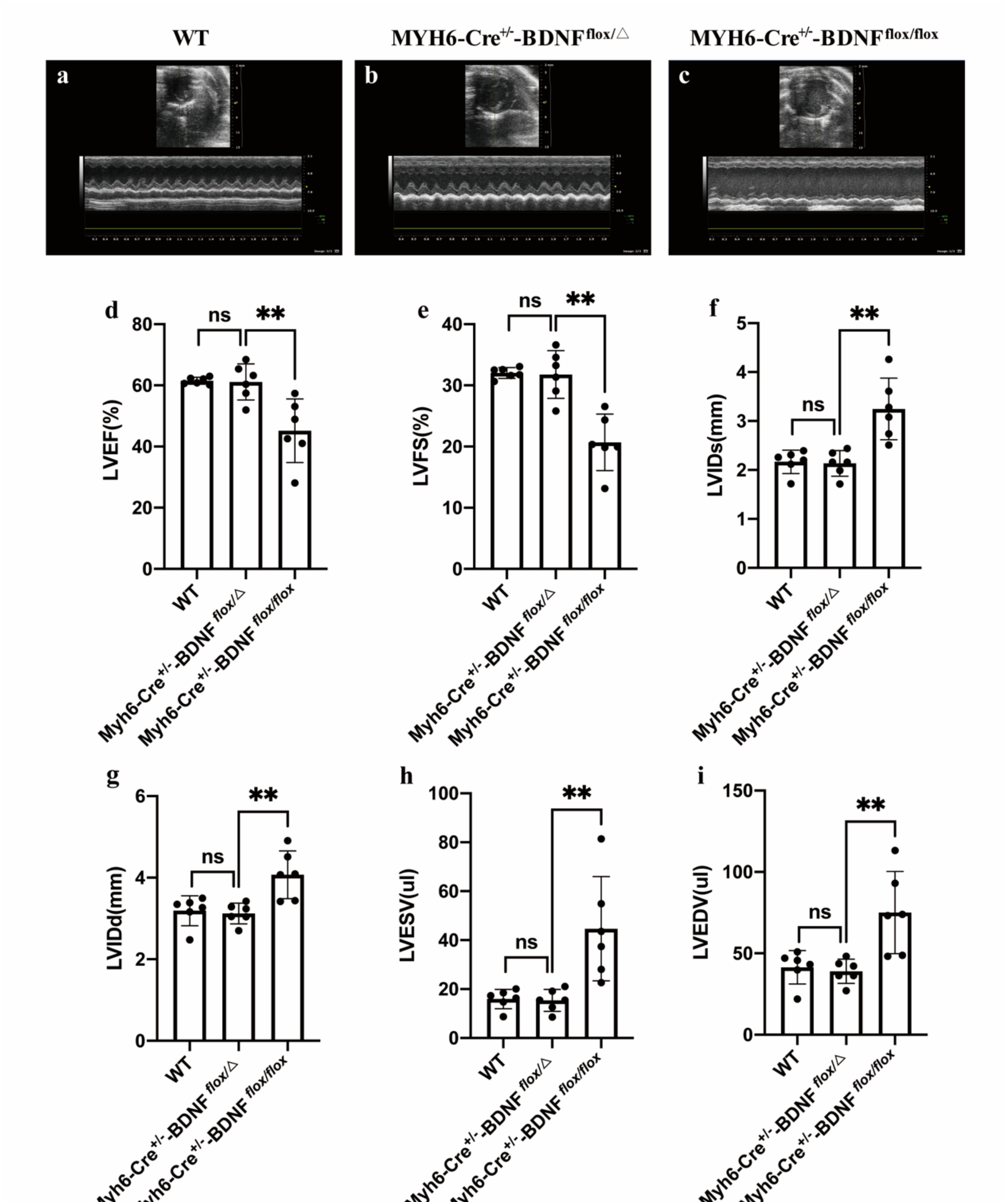

Supplementary Figure 31. There was no significant difference of cardiac function of left ventricular geometric parameters between 3-month-old MYH6-Cre$^{+/-}$-BDNF$^{flox/\triangle}$ myocardium and 3-month-old WT myocardium. However, cardiac dysfunction was found in 3-month-old MYH6-Cre$^{+/-}$-BDNF$^{flox/flox}$ heart. a: Representative echocardiography of 3-month-old WT hearts. **b:** Representative

echocardiography of 3-month-old MYH6-$Cre^{+/-}$-$BDNF^{flox/\triangle}$ hearts. **c:** Representative echocardiography of 3-month-old MYH6-$Cre^{+/-}$-$BDNF^{flox/flox}$ hearts. Echocardiography showed that the left ventricular ejection fraction (LVEF) (d) and left ventricular fractional shortening (LVFS) (e) of in 3-month-old MYH6-$Cre^{+/-}$-$BDNF^{flox/flox}$ hearts were significantly lower than those of 3-month-old MYH6-$Cre^{+/-}$-$BDNF^{flox/\triangle}$ and 3-month-old WT hearts, while the left ventricular end-systolic internal diameter (LVIDs) (f), left ventricular end-diastolic internal diameter (LVIDd) (g), left ventricular end-systolic volume (LVESV) (h) and left ventricular end-diastolic volume (LVEDV) (i) of 3-month-old MYH6-$Cre^{+/-}$-$BDNF^{flox/flox}$ hearts were significantly higher than those of 3-month-old MYH6-$Cre^{+/-}$-$BDNF^{flox/\triangle}$ and 3-month-old WT hearts. All the above parameters of 3-month-old MYH6-$Cre^{+/-}$-$BDNF^{flox/\triangle}$ hearts were similar to 3-month-old WT hearts. **: $p<0.01$. n=6. Taking the consideration of that MYH6-$Cre^{+/-}$ mice, MYH6-$Cre^{+/-}$-$BDNF^{flox/\triangle}$ mice and MYH6-$Cre^{+/-}$-$BDNF^{flox/flox}$ mice are same as MYH6-$Cre^{+/-}$ heterozygous, while, the pathological phenotypes and dysfunction of cardiac function are found in MYH6-$Cre^{+/-}$-$BDNF^{flox/flox}$ hearts but not in MYH6-$Cre^{+/-}$-$BDNF^{flox/\triangle}$ hearts and WT hearts. Therefore, the results of supplementary Figure 1-4 clearly demonstrated that pathological phenotypes and dysfunction of cardiac function found in present study from our established MYH6-$Cre^{+/-}$-$BDNF^{flox/flox}$ line were attributed to the ablation of BDNF but not the potential cardiotoxicity of Myh6-Cre.

Supplementary Table 1. IPA analysis identifies increase of weight gain in MYH6-Cre-BDNF$^{-/-}$ heart (activation z-score=2.53; p=7.67E-05).

Genes	ID	Expr Log Ratio	Phospho False Discovery	Location
CIDEC	ENSMUSG00000030278	↑ 2.304	2.35E-11	Cytoplasm
GCK	ENSMUSG00000041798	↑ 1.425	2.12E-03	Cytoplasm
MOGAT1	ENSMUSG00000012187	↑ ∞	3.18E-01	Cytoplasm
NCF1	ENSMUSG00000015950	↑ 0.688	2.77E-01	Cytoplasm
NPY2R	ENSMUSG00000028004	↑ ∞	4.44E-01	Plasma Membrane
NTRK2	ENSMUSG00000055254	↑ 0.905	1.19E-01	Plasma Membrane
PRKAR2B	ENSMUSG00000002997	↑ 1.072	4.11E-01	Cytoplasm
PTTG1	ENSMUSG00000020415	↑ 3.043	3.85E-25	Nucleus
SCD	ENSMUSG00000037071	↑ 2.001	7.42E-03	Cytoplasm

Supplementary Table 2. IPA analysis identifies increase of synthesis of fatty acid in MYH6-Cre-BDNF$^{-/-}$ heart (activation z-score=2.231; p=4.47E-07).

Genes	ID	Expr Log Ratio	Phospho False Discovery	Location
ACACA	ENSMUSG00000020532	↑ 0.590	2.85E-01	Cytoplasm
ALOX5AP	ENSMUSG00000060063	↑ 1.100	4.45E-02	Plasma Membrane
APOC2	ENSMUSG00000002992	↑ ∞	1.52E-01	Extracellular Space
CYP2E1	ENSMUSG00000025479	↑ 1.893	1.70E-08	Cytoplasm
EGF	ENSMUSG00000028017	↑ 0.834	9.48E-02	Extracellular Space
FASN	ENSMUSG00000025153	↑ 1.524	4.21E-03	Cytoplasm
HBB	ENSMUSG00000052305	↓ -0.740	2.93E-01	Cytoplasm
LTF	ENSMUSG00000032496	↑ 3.135	2.34E-01	Extracellular Space
NCF1	ENSMUSG00000015950	↑ 0.688	2.77E-01	Cytoplasm
PTAFR	ENSMUSG00000056529	↑ 0.941	4.21E-01	Plasma Membrane
RAC1	ENSMUSG00000001847	↑ 1.404	6.32E-02	Plasma Membrane
SCD	ENSMUSG00000037071	↑ 2.001	7.42E-03	Cytoplasm

Supplementary Table 3. IPA analysis identifies increase of synthesis of lipid in MYH6-Cre-BDNF$^{-/-}$ heart (activation z-score=2.32; p=4.63E-06).

Genes	ID	Expr Log Ratio	Phospho False Discovery	Location
ACACA	ENSMUSG00000020532	↑ 0.590	2.85E-01	Cytoplasm
ALOX5AP	ENSMUSG00000060063	↑ 1.100	4.45E-02	Plasma Membrane
AMH	ENSMUSG00000035262	↓-2.853	3.18E-01	Extracellular Space
APOC2	ENSMUSG00000002992	↑ ∞	1.52E-01	Extracellular Space
B4GALNT1	ENSMUSG00000006731	↑ 1.130	1.58E-01	Cytoplasm
CYP2E1	ENSMUSG00000025479	↑ 1.893	1.70E-08	Cytoplasm
EGF	ENSMUSG00000028017	↑ 0.834	9.48E-02	Extracellular Space
FASN	ENSMUSG00000025153	↑ 1.524	4.21E-03	Cytoplasm
HBB	ENSMUSG00000052305	↓ -0.740	2.93E-01	Cytoplasm
LPIN1	ENSMUSG00000020593	↑ 1.242	1.00E-01	Nucleus
LTF	ENSMUSG00000032496	↑ 3.135	2.34E-01	Extracellular Space
NCF1	ENSMUSG00000015950	↑ 0.688	2.77E-01	Cytoplasm
PTAFR	ENSMUSG00000056529	↑ 0.941	4.21E-01	Plasma Membrane
RAC1	ENSMUSG00000001847	↑ 1.404	6.32E-02	Plasma Membrane
RDH16	ENSMUSG00000056148	↑ 2.225	3.71E-01	Cytoplasm
RETN	ENSMUSG00000012705	↑ 1.871	2.44E-01	Extracellular Space
SCD	ENSMUSG00000037071	↑ 2.001	7.42E-03	Cytoplasm

Supplementary Table 4. IPA analysis identifies increase of biosynthesis of polyunsaturated fatty acid in MYH6-Cre-BDNF$^{-/-}$ heart (activation z-score=2.40; p=1.25E-04).

Genes	ID	Expr Log Ratio	Phospho False Discovery	Location
ADIPOQ	ENSMUSG00000022878	↑ 1.964	1.08E-05	Extracellular Space
ALOX5AP	ENSMUSG00000060063	↑ 1.100	4.45E-02	Plasma Membrane
EGF	ENSMUSG00000028017	↑ 0.834	9.48E-02	Extracellular Space
FASN	ENSMUSG00000025153	↑ 1.524	4.21E-03	Cytoplasm
HBB	ENSMUSG00000052305	↓ -0.740	2.93E-01	Cytoplasm
LTF	ENSMUSG00000032496	↑ 3.135	2.34E-01	Extracellular Space
NCF1	ENSMUSG00000015950	↑ 0.688	2.77E-01	Cytoplasm
PTAFR	ENSMUSG00000056529	↑ 0.941	4.21E-01	Plasma Membrane
RAC1	ENSMUSG00000001847	↑ 1.404	6.32E-02	Plasma Membrane

Supplementary Table 5. IPA analysis identifies increase of synthesis of eicosanoid in MYH6-Cre-BDNF$^{-/-}$ heart (activation z-score=2.231; p=4.47E-07).

Genes	ID	Expr Log Ratio	Phospho False Discovery	Location
ADIPOQ	ENSMUSG00000022878	↑ 1.964	1.08E-05	Extracellular Space
ALOX5AP	ENSMUSG00000060063	↑ 1.100	4.45E-02	Plasma Membrane
EGF	ENSMUSG00000028017	↑ 0.834	9.48E-02	Extracellular Space
FASN	ENSMUSG00000025153	↑ 1.524	4.21E-03	Cytoplasm
HBB	ENSMUSG00000052305	↓ -0.740	2.93E-01	Cytoplasm
LTF	ENSMUSG00000032496	↑ 3.135	2.34E-01	Extracellular Space
NCF1	ENSMUSG00000015950	↑ 0.688	2.77E-01	Cytoplasm
PTAFR	ENSMUSG00000056529	↑ 0.941	4.21E-01	Plasma Membrane
RAC1	ENSMUSG00000001847	↑ 1.404	6.32E-02	Plasma Membrane

Supplementary Table 6. IPA analysis identifies increase of concentration of colfosceril palmitate in MYH6-Cre-BDNF$^{-/-}$ heart (activation z-score=2.00; p=2.35E-06).

Genes	ID	Expr Log Ratio	Phospho False Discovery	Location
ACACA	ENSMUSG00000020532	↑ 0.590	2.85E-01	Cytoplasm
FASN	ENSMUSG00000025153	↑ 1.524	4.21E-03	Cytoplasm
INSIG1	ENSMUSG00000045294	↑ 0.765	2.89E-01	Cytoplasm
SCD	ENSMUSG00000037071	↑ 2.001	7.42E-03	Cytoplasm

Supplementary Table 7. IPA analysis identifies increase of concentration of triacylglycerol in MYH6-Cre-BDNF$^{-/-}$ heart (activation z-score=2.25; p=5.74E-06).

Genes	ID	Expr Log Ratio	Phospho False Discovery	Location
ACACA	ENSMUSG00000020532	↑ 0.590	2.85E-01	Cytoplasm
CAV1	ENSMUSG00000007655	↓-0.872	1.81E-02	Plasma Membrane
CIDEA	ENSMUSG00000024526	↑ 0.717	4.46E-01	Cytoplasm
CXCL14	ENSMUSG00000021508	↑ 0.718	3.33E-01	Extracellular Space
EGF	ENSMUSG00000028017	↑ 0.834	9.48E-02	Extracellular Space
FASN	ENSMUSG00000025153	↑ 1.524	4.21E-03	Cytoplasm

INSIG1	ENSMUSG00000045294	↑ 0.765	2.89E-01	Cytoplasm
KLB	ENSMUSG00000029195	↑ 2.142	5.83E-02	Plasma Membrane
NCOA5	ENSMUSG00000039804	↓-0.700	1.16E-01	Nucleus
PCK1	ENSMUSG00000027513	↑ 1.553	5.98E-02	Cytoplasm
PRKAR2B	ENSMUSG00000002997	↑ 1.072	4.11E-01	Cytoplasm
RETN	ENSMUSG00000012705	↑ 1.871	2.44E-01	Extracellular Space
SCD	ENSMUSG00000037071	↑ 2.001	7.42E-03	Cytoplasm

Supplementary Table 8. IPA analysis identifies increase of fatty acid metabolism in MYH6-Cre-BDNF$^{-/-}$ heart (activation z-score=2.26; p=2.46E-10).

Genes	ID	Expr Log Ratio	Phospho False Discovery	Location
ABCC2	ENSMUSG00000025194	↑ 2.582	3.48E-01	Plasma Membrane
ACACA	ENSMUSG00000020532	↑ 0.590	2.85E-01	Cytoplasm
ADIPOQ	ENSMUSG00000022878	↑ 1.964	1.08E-05	Extracellular Space
ALOX5AP	ENSMUSG00000060063	↑ 1.100	4.45E-02	Plasma Membrane
APOC2	ENSMUSG00000002992	↑ ∞	1.52E-01	Extracellular Space
B4GALNT1	ENSMUSG00000006731	↑ 1.130	1.58E-01	Cytoplasm
CYP2E1	ENSMUSG00000025479	↑ 1.893	1.70E-08	Cytoplasm
EGF	ENSMUSG00000028017	↑ 0.834	9.48E-02	Extracellular Space
FASN	ENSMUSG00000025153	↑ 1.524	4.21E-03	Cytoplasm
HBB	ENSMUSG00000052305	↓ -0.740	2.93E-01	Cytoplasm
LBP	ENSMUSG00000016024	↑ 1.179	7.42E-03	Plasma Membrane
LTF	ENSMUSG00000032496	↑ 3.135	2.34E-01	Extracellular Space
NCF1	ENSMUSG00000015950	↑ 0.688	2.77E-01	Cytoplasm
PLAAT1	ENSMUSG00000022525	↑ 2.056	6.20E-05	Cytoplasm
PTAFR	ENSMUSG00000056529	↑ 0.941	4.21E-01	Plasma Membrane
RAC1	ENSMUSG00000001847	↑ 1.404	6.32E-02	Plasma Membrane
UCP1	ENSMUSG00000031710	↑ 4.666	2.63E-02	Cytoplasm
SCD	ENSMUSG00000037071	↑ 2.001	7.42E-03	Cytoplasm

Supplementary Table 9. IPA analysis identifies activation of STAT3 pathway in MYH6-Cre-BDNF$^{-/-}$ heart (activation z-score=2; p=2.36E-02).

Genes	ID	Expr Log Ratio	Phospho False Discovery	Location
CXCR2	ENSMUSG00000000261	↑ 2.549	2.00E-01	Plasma Membrane
EGF	ENSMUSG00000028017	↑ 0.834	9.48E-02	Extracellular Space
IFNLR1	ENSMUSG00000062157	↑ 0.652	4.27E-01	Plasma Membrane
IL2RB	ENSMUSG00000068227	↑ 1.562	4.44E-01	Plasma Membrane
NTRK2	ENSMUSG00000055254	↑ 0.905	1.19E-01	Plasma Membrane
RAC1	ENSMUSG00000001847	↑ 1.404	6.32E-02	Plasma Membrane

Supplementary Table 10. IPA analysis identifies activation of melatonin degradation I (activation z-score=2; p=1.66E-02) and acetone degradation I (activation z-score=2; p=1.37E-03) in MYH6-Cre-BDNF$^{-/-}$ heart.

Genes	ID	Expr Log Ratio	Phospho False Discovery	Location
CXCR2	ENSMUSG00000000261	↑ 2.549	2.00E-01	Plasma Membrane
EGF	ENSMUSG00000028017	↑ 0.834	9.48E-02	Extracellular Space
IFNLR1	ENSMUSG00000062157	↑ 0.652	4.27E-01	Plasma Membrane
IL2RB	ENSMUSG00000068227	↑ 1.562	4.44E-01	Plasma Membrane

Supplementary Table 11-20. IPA revealed that 41 inflammation regulatory genes (upregulated: 38 genes; downregulated: 3 genes) were involved in regulating the increase in inflammatory activity in MYH6-Cre-BDNF$^{-/-}$ hearts.

Gene	Expr Log Ratio	Phospho False Discovery	**Table-S11** Activation of Inflammation response	**Table-S12** Activation of Recruitment of phagocytes	**Table-S13** Activation of Chemotaxis of neutrophils	**Table-S14** Activation of Chemotaxis of granulocytes	**Table-S15** Activation of Chemotaxis of leukocytes	**Table-S16** Activation of leukocytes	**Table-S17** Activation of macrophages	**Table-S18** Activation of mononuclear leukocytes	**Table-S19** Activation of phagocytes	**Table-S20** Activation of lymphocytes
SERPINA1	↑ ∞	4.78E-01	+		+	+	+	+				
MUC5B	↑ 8.334	8.31E-06	+						+		+	
CLCA1	↑ 6.276	4.41E-03							+		+	
S100A9	↑ 3.872	5.61E-02	+		+	+	+	+		+		+
LTF	↑ 3.135	2.34E-01	+						+	+	+	+
CCL5	↑ 2.969	4.72E-01	+	+	+	+	+	+	+	+	+	+
IGHM	↑ 2.898	1.49E-01								+		+
Chil3/Chil4	↑ 2.810	1.82E-01	+			+	+	+	+		+	
S100A8	↑ 2.748	1.82E-01	+		+	+	+	+				
HP	↑ 2.724	1.94E-06	+				+	+				
Ccl27a	↑ 2.723	8.98E-09	+					+				
CXCR2	↑ 2.549	2.00E-01	+	+	+	+	+	+				
Ear2	↑ 2.309	8.94E-04	+				+	+				
ACKR1	↑ 2.302	7.37E-04		+								
ADIPOQ	↑ 1.964	1.08E-05		+								
Retnla	↑ 1.727	6.23E-04	+						+	+	+	+
RAC1	↑ 1.404	6.32E-02	+	+	+	+	+	+				
LCN2	↑ 1.344	4.48E-01	+	+	+	+	+	+	+	+	+	
ZBP1	↑ 1.280	3.51E-03	+									
SASH3	↑ 1.250	2.69E-03								+		+
ITGAM	↑ 1.227	4.75E-03									+	
LBP	↑ 1.179	7.42E-03	+		+	+	+	+	+	+	+	
NR4A3	↑ 1.153	9.29E-03								+		+
Gm21596/Hmgb1	↑ 1.141	3.73E-04		+							+	
LYZ	↑ 1.139	4.23E-01		+								
VAMP8	↑ 1.123	1.23E-06								+		+
ALOX5AP	↑ 1.100	4.45E-02	+	+								
ITGB2	↑ 1.085	9.79E-03		+							+	
THBS4	↑ 1.010	2.13E-03	+		+	+	+	+				
TREM2	↑ 0.953	7.77E-03	+					+	+	+	+	
PTAFR	↑ 0.941	4.21E-01	+									
SLC1A5	↑ 0.904	1.72E-04	+									
C3AR1	↑ 0.748	9.39E-03	+	+	+	+	+	+				+
CD38	↑ 0.728	1.62E-03	+				+	+		+		+
CXCL14	↑ 0.718	3.33E-01	+		+	+	+	+				
F13A1	↑ 0.712	2.60E-04		+								
Tlr13	↑ 0.689	6.95E-03	+									
PLP1	↑ 0.627	4.49E-01										+
BCL6	↓-0.686	3.50E-03	+									
ANGPT1	↓-0.815	3.18E-01							+		+	
SLFN12L	↓-1.109	1.17E-03		+								

DOI [to be added]
